# Herbal Medicine for Cardiovascular Diseases: Efficacy, Mechanisms, and Safety

**DOI:** 10.3389/fphar.2020.00422

**Published:** 2020-04-07

**Authors:** Abdullah Shaito, Duong Thi Bich Thuan, Hoa Thi Phu, Thi Hieu Dung Nguyen, Hiba Hasan, Sarah Halabi, Samar Abdelhady, Gheyath K. Nasrallah, Ali H. Eid, Gianfranco Pintus

**Affiliations:** ^1^Department of Biological and Chemical Sciences, Lebanese International University, Beirut, Lebanon; ^2^Department of Biochemistry, University of Medicine and Pharmacy, Hue University, Hue City, Vietnam; ^3^Department of Physiology, University of Medicine and Pharmacy, Hue University, Hue City, Vietnam; ^4^Institute of Anatomy and Cell Biology, Justus Liebig University Giessen, Giessen, Germany; ^5^Biology Department, Faculty of Arts and Sciences, American University of Beirut, Beirut, Lebanon; ^6^Faculty of Medicine, Alexandria University, Alexandria, Egypt; ^7^Department of Biomedical Sciences, College of Health Sciences, Qatar University, Doha, Qatar; ^8^Department of Pharmacology and Toxicology, American University of Beirut, Beirut, Lebanon; ^9^Department of Medical Laboratory Sciences, University of Sharjah, Sharjah, United Arab Emirates; ^10^Department of Biomedical Sciences, Faculty of Medicine, University of Sassari, Sassari, Italy

**Keywords:** herbal medicine, cardiovascular diseases, atherosclerosis, hypertension, medicinal plants, antioxidants, oxidative stress, inflammation

## Abstract

Cardiovascular diseases (CVDs) are a significant health burden with an ever-increasing prevalence. They remain the leading causes of morbidity and mortality worldwide. The use of medicinal herbs continues to be an alternative treatment approach for several diseases including CVDs. Currently, there is an unprecedented drive for the use of herbal preparations in modern medicinal systems. This drive is powered by several aspects, prime among which are their cost-effective therapeutic promise compared to standard modern therapies and the general belief that they are safe. Nonetheless, the claimed safety of herbal preparations yet remains to be properly tested. Consequently, public awareness should be raised regarding medicinal herbs safety, toxicity, potentially life-threatening adverse effects, and possible herb–drug interactions. Over the years, laboratory data have shown that medicinal herbs may have therapeutic value in CVDs as they can interfere with several CVD risk factors. Accordingly, there have been many attempts to move studies on medicinal herbs from the bench to the bedside, in order to effectively employ herbs in CVD treatments. In this review, we introduce CVDs and their risk factors. Then we overview the use of herbs for disease treatment in general and CVDs in particular. Further, data on the ethnopharmacological therapeutic potentials and medicinal properties against CVDs of four widely used plants, namely *Ginseng*, *Ginkgo biloba*, *Ganoderma lucidum*, and *Gynostemma pentaphyllum*, are gathered and reviewed. In particular, the employment of these four plants in the context of CVDs, such as myocardial infarction, hypertension, peripheral vascular diseases, coronary heart disease, cardiomyopathies, and dyslipidemias has been reviewed, analyzed, and critically discussed. We also endeavor to document the recent studies aimed to dissect the cellular and molecular cardio-protective mechanisms of the four plants, using recently reported *in vitro* and *in vivo* studies. Finally, we reviewed and reported the results of the recent clinical trials that have been conducted using these four medicinal herbs with special emphasis on their efficacy, safety, and toxicity.

## Introduction

Cardiovascular diseases (CVDs) are diseases of the heart or blood vessels. CVDs register a global annual toll of more than 17 million deaths. As a result, CVDs remain the world's most common cause of death and are a major economic and health burden, worldwide. The World Health Organization (WHO) reported that CVDs account for 31% of annual global deaths (World Health Organization, 2017). In Europe, CVDs account for 45% of all deaths according to the European Cardiovascular Disease Statistics 2017 (Martinet et al., 2019). The American Heart Association's current statistics estimate that around half of the population of the USA has a form of CVD (Benjamin et al., 2019).

**Figure 1:**
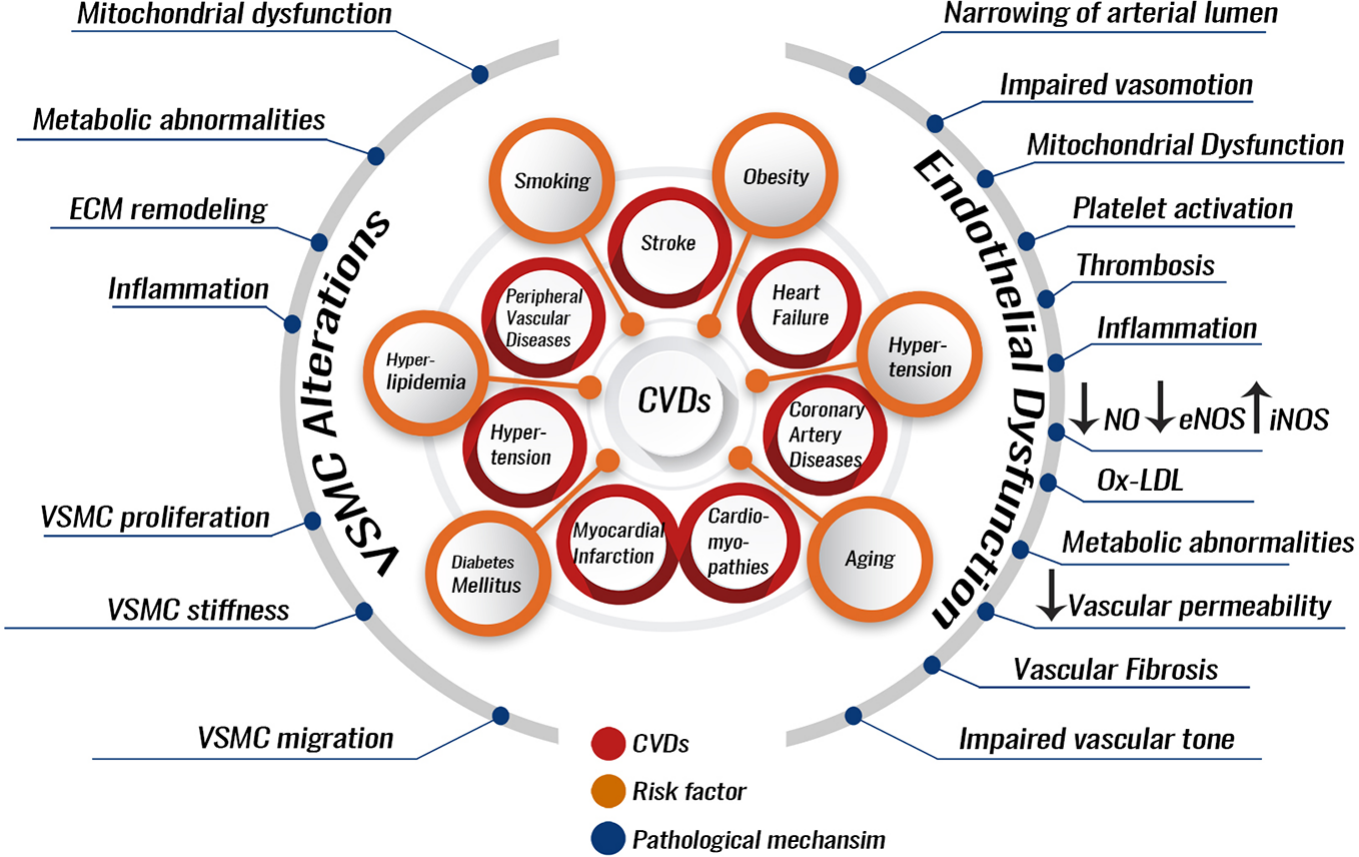
Pathological processes involved in the development and progression of CVDs. Several risk factors can predispose to CVDs. These can include hypertension, smoking, dyslipidemia stemming from an unhealthy diet, or endocrinopathies like diabetes mellitus, hypothyroidism, and aging. The risk factors can lead to pathological alterations most of which can be due to endothelial dysfunction or VSMC alterations. Endothelial dysfunction or VSMC alterations increase the risk of developing atherosclerosis and hypertension. Atherosclerosis and hypertension are themselves CVDs risk factors and enhancers for the development of other CVDs like myocardial infarction, coronary artery diseases, or stroke. VSMC, vascular smooth muscle cell; ECM, extracellular matrix; NO, nitric oxide; eNOS, endothelial nitric oxide synthase; iNOS, inducible nitric oxide synthase; Ox-LDL, oxidized low-density lipoprotein.

CVDs are a variety of diseases including peripheral vascular diseases, coronary heart disease (CHD), heart failure, heart attack (myocardial infarction), stroke, cardiomyopathies, dyslipidemias, and hypertension, among others (**Figure 1**) (Toth, 2007; Reiner et al., 2019). CVDs majorly originate from a vascular dysfunction, which then leads to organ damage. For example, the heart can suffer a heart attack, or the brain can suffer a stroke due to vascular impairment. Major culprits in vascular impairment include atherosclerosis, thrombosis, and high blood pressure (BP). Common risk factors for CVDs include smoking, unhealthy diet, diabetes mellitus, hyperlipidemia, elevated levels of low-density lipoprotein cholesterol (LDL), suppressed levels of high-density lipoprotein cholesterol (HDL), and hypertension (**Figure 1**) (World Health Organization, 2017).

CVDs prevention is favored by a healthy vascular endothelium. A healthy endothelium exhibits vasodilatory, anti-atherogenic, and anti-inflammatory properties (Celermajer, 1997). Several risk factors for CVDs lead to endothelial cell (EC) dysfunction, which has been implicated as a key event in the pathogenesis of atherosclerosis, coronary vasoconstriction, and, probably, myocardial ischemia. Interestingly, EC dysfunction is a reversible phenomenon, which opens the door for CVD therapies based on its reversion (**Figure 1**) (Celermajer, 1997).

Recently, inflammation has been confirmed as a risk factor for CVDs, especially during atherosclerosis and coronary artery disease. High levels of high-sensitivity C-reactive protein (hs-CRP) and/or interleukin-6 (IL-6) are associated with higher absolute cardiovascular risk (Ridker et al., 1997; Ridker et al., 2000), where the CANTOS study, for the first time, established reduced rates of cardiovascular events following an anti-interleukin-1 beta (IL-1β) based therapy, independent of cholesterol levels (Ridker et al., 2017). Furthermore, common CVDs risk factors, such as diabetes or hypertension, can predispose to CVDs by the mediation of inflammation (Dokken, 2008; Aday and Ridker, 2018).

In the case of atherosclerosis, for example, inflammation can cause EC functional impairment. Dysfunctional ECs allow the accumulation of low-density lipoprotein (LDL) particles in the vessel wall intima where they become modified into oxidized LDL. Oxidized LDL can then activate the dysfunctional ECs to expose cell adhesion molecules (VCAM-1 and ICAM-1) that bind to and recruit inflammatory leukocytes (T-cells and monocytes) into the subendothelial space (Davies et al., 1993; Moore and Tabas, 2011). These inflammatory blood cells secrete interleukins and cytokines, produce reactive oxygen species (ROS) and thus form an inflamed microenvironment within the arterial wall. The inflamed microenvironment promotes vascular smooth muscle cell (VSMC) proliferation, matrix build-up, and lipid deposition, leading to the formation of an atherosclerotic plaque. The monocytes can reach the intima of the vessel, differentiate into macrophages, and uptake oxidized LDL to become foam cells (Tabas et al., 2007; Moore and Tabas, 2011; Douglas and Channon, 2014; Saleh Al-Shehabi et al., 2016; Martinet et al., 2019). Gradual intimal thickening takes place over the years and continues to expand causing decreased or complete occlusion of blood flow to organs, ultimately resulting in CVDs, such as myocardial infarction or stroke (Maguire et al., 2019). In addition, VSMCs proliferation leads to narrowing of the arterial lumen and dysregulation of the vasotone (Douglas and Channon, 2014). Usually several atherosclerotic plaques form in the intima and one of these may end up undergoing a necrotic breakdown, leading to acute luminal thrombosis, blood vessel occlusion, and cardiovascular complications, including myocardial infarction, unstable angina (chest pain caused by heart muscle ischemia), sudden cardiac death, or a stroke (Virmani et al., 2002). As a result, atherosclerosis is not only a risk factor but also a major contributor to CVD incidence. Around 50% of all deaths in developed countries are due to atherosclerosis (Tedgui and Mallat, 2006).

Hypertension also referred to as high BP, is a CVD and a major risk factor and contributor to other CVDs and other diseases (2017). Hypertension is an independent predisposing factor for heart failure, coronary artery disease, stroke, retinopathy, nephropathy, and peripheral arterial diseases (Sawicka et al., 2011; NCD Risk Factor Collaboration, 2017). Most of these diseases are associated with high mortality and morbidity (Abegaz et al., 2017). Additionally, hypertension is the single most significant risk factor for atherosclerosis, and any clinical outcome of atherosclerosis thereof (Sawicka et al., 2011). Hypertension is a “silent killer” as it does not show symptoms until later stages of the disease (Sawicka et al., 2011). Because of this, it is not surprising that hypertension affects 1.4 billion people and accounts for about 9.4 million deaths per year (Cooper et al., 2017; Egan et al., 2019). Lastly, hypertension prevalence is estimated to have a 30% worldwide increase by 2025 (Kearney et al., 2005).

The American Heart Association Hypertension Guidelines define hypertension as a persistent elevation of BP in the arteries [systolic BP (SBP) higher than 130/diastolic BP (DBP) higher than 80 mm Hg] (Muntner et al., 2018). If an elevated BP is left unmanaged, it can induce arterial remodeling; the walls of small vessels thicken, and the vessels lose their elasticity and become narrower. This process is called arteriosclerosis and can lead to “target organ damage” (TOD) (Triantafyllidi et al., 2010; Fan et al., 2017). TOD affects several organs such as the brain, kidney, or retina and may lead to death (Mensah, 2016; Abegaz et al., 2017). Arteriosclerosis can be witnessed in coronary vessels where it may cause a myocardial infarction (Rakugi et al., 1996). In the brain, arteriosclerosis can cause vessel lumen narrowing, vessel wall hardening, and blood clot formation, potentially causing a stroke (Johansson, 1999). Strokes have effects on cognitive and physical behaviors, and may result in dementia, paralysis, or death (Abegaz et al., 2017). Nephrosclerosis of the kidney is also due to arteriosclerosis which stiffens the nephron, ultimately affecting renal filtration and causing electrolyte imbalances (Bidani and Griffin, 2004; Lim et al., 2016). Overall, TOD proceed through hypertension-induced microvascular injuries in the cases of retinopathy and nephropathy and through hypertension-induced macrovascular injuries in the cases of stroke and myocardial infarctions (Nadar, 2011).

BP regulation, and therefore hypertension, depends on two main factors: cardiac output and systemic vascular resistance. Increased cardiac output or vascular resistance elevate BP. Cardiac output is majorly affected by sodium intake, renal function, and mineralocorticoids. Vascular resistance is affected by the sympathetic nervous system (SNS), rennin–angiotensin system (RAS), humoral factors, and local autoregulation (DiBona, 2013). SNS and RAS exert their effects mainly by eliciting vasoconstriction and inducing sodium retention. Humoral mediators can be vasoconstrictors like endothelin, angiotensin II, catecholamines, or vasodilators, for instance nitric oxide (NO), prostaglandins, and kinins (Oparil et al., 2003). Other factors that can modulate BP include blood flow velocity, blood viscosity, vascular wall stiffness, oxidative stress of VSMCs or ECs, VSMCs proliferation and shape changes, and EC health (Rodrigo et al., 2011).

Despite advances in CVD management and treatment, CVDs still claim more lives than the combination of all cancer forms (Mozaffarian et al., 2015). As a result, in recent years there has been major enforcement on CVD prevention (Reiner et al., 2019). Therefore, new treatment options are urgently warranted for all types of CVDs, considering the continued burden stemming from CVDs is still substantial.

## Herbal and Plant Products as Medicinal Drugs

Traditional medicine and ethnomedicine, defined as the study of the traditional medicines practiced by various ethnic groups, are as old as human history. Traditional medicine historically relied on natural resources as medications. Historically, herbs, generally defined as any form of plant or plant product (Tachjian et al., 2010), and plant extracts formed the basis of the first drugs used in traditional medicine systems of many cultures and civilizations. Plants and herbs have always been a common source of medications, either in the form of traditional extracts or as pure active compounds (Fabricant and Farnsworth, 2001). Evidently, nature is a very important source for finding new drugs that leads to the treatment of diseases. Famous drugs from herbal and plant sources include aspirin from the *Salix alba* L. tree, digoxin (cardiac glycoside) from *Digitalis purpurea*, ephedrine from *Ephedra sinica*, lovastatin from *Monascus purpureus* L., taxol from *Taxus brevifolia*, reserpine from *Rauvolfia serpentina*, and many others (Harvey, 2000; Frishman et al., 2009; Cragg and Newman, 2013). Interestingly, reserpine is still an effective treatment for hypertension (Weber et al., 2014). Notably, the discovery of antimalarial drugs, quinine from the bark of *Cinchona* species and artemisinin from *Artemisia annua* L., represent a typical example of how ethnomedicine can guide drug discovery (Cragg and Newman, 2013).

The earliest records of drugs of natural origin, found in Mesopotamia (from around 2600 BCE), describe the use of approximately 1000 plant-derived compounds. The best record of using natural extracts in therapy is the Egyptians' Ebers Papyrus (from 1500 BCE), which documents more than 700 natural drugs, mainly of plant origin. The Chinese Materia Medica record (BCE 1100) describes 52 natural medicinal preparations, and the Indian Ayurvedic record (BCE 1000) describes more than 800 natural medicinal extracts (Cragg and Newman, 2013; Otvos et al., 2019). Hippocrates also applied phytotherapy, or healing with herbs, in his treatments (Otvos et al., 2019).

In 1985 WHO estimated that around 65% of the world population mostly depended on plant-derived traditional medicines (Farnsworth et al., 1985). People in different countries have come to use identical or comparable plants or herbal preparations for the prevention and/or treatment of physical and mental illnesses. Traditional Medicine Centers of the WHO identified 122 compounds to be commonly used in the Center's host countries. Interestingly, the 122 compounds have been reported to derive from only 94 plant species and are used for similar ethnomedical treatments in the different host countries (Farnsworth et al., 1985). Examples of such compounds include galegine, from *Galega officinalis* L., the base for the synthesis of metformin and similar bisguanidine-type antidiabetic drugs, and papaverine from *Papaver somniferum* which is the base for making the antihypertensive drug verapamil (Fabricant and Farnsworth, 2001). Commercially, drug production from natural products such as herbs is a viable commodity, where 39% of the 520 new drugs approved between 1983 and 1994 were natural compounds or derived from natural compounds and 60–80% of antibacterial and anticancer drugs were derived from natural products in that same period (Harvey, 2000).

Despite the many successes of using natural products for drug production, advances in combinatorial chemistry (in the late 1980s) shifted the focus of drug discovery efforts from natural products to synthesis at the laboratory bench (Cragg and Newman, 2013). This is mainly because natural product-based drug discovery and development is a complex endeavor demanding costly and highly integrated interdisciplinary approaches (Davison and Brimble, 2019; Otvos et al., 2019). Nonetheless, currently the use of natural products as drugs or as drug discovery platforms is “well and alive” (Newman and Cragg, 2016). In fact, traditional herbal and plant-derived extracts are becoming main stream as advances in scientific research are showing their importance in the prevention and treatment of diseases (Frishman et al., 2009).

Numerous and chemically diverse secondary metabolites have been purified from plant bioactives and have been optimized for exerting a biological effect, nonetheless, they are still away from exhaustive investigation for clinical use. However, recent published scientific evidence, technological advances, and research trends clearly point that naturally-derived compounds will be major sources of new drugs (Davison and Brimble, 2019; Otvos et al., 2019). This has provided a driving cause for the renewed popularity of traditional herbal and plant-derived medications among researchers, despite developments in combinatorial chemical synthesis and the production of modern synthetic drugs (Frishman et al., 2009).

Another reason for the regained interest in medicinal plant products is that, in their attempts to control diseases amid scarce socioeconomic resources, rural communities in developing countries have found resort in traditional herbal and plant-derived remedies. This is due to several factors, but in particular to the fact that plant-based medicines are a cheaper alternative with fewer side effects (Frishman et al., 2009; Tabassum and Ahmad, 2011). Herbal and plant remedies are not only economical, but they also contain thousands of bioactive components that have known therapeutic applications (Pan et al., 2013). Additionally, because herbs are viewed as food products, they are not subject to the same surveillance and regulation as conventional drugs. Moreover, herbal remedies are viewed by patients as being natural and therefore safe. However, more research efforts are required to validate the efficacy and the safety profile of such medicaments since many have adverse outcomes that can sometimes have life-threatening effects. It should also be noted that there is concern regarding herb–drug interaction (Tachjian et al., 2010; Anwar et al., 2016; Yuan et al., 2016).

One more reason for the revived interest in natural products is that the biological activity and structural diversity of natural products are unmatched by any available synthetic drug screening library (Davison and Brimble, 2019). Natural products have been selected by nature for bioactivity, over millions of years. As a result, natural product screening libraries need not be superfluously big, as is the case with synthetic drug screening libraries. In addition, natural products need only minor structural changes to optimize their drugability (Harvey, 2000; Gerry and Schreiber, 2018). As such, natural products offer “privileged scaffolds” and serve as biologically “pre-validated platforms” for the design of compound candidate drug libraries (Davison and Brimble, 2019). Recent progress has focused on improving the potency, selectivity, and pharmacokinetics of bioactive natural products through structural modifications, which has led to the production of novel drug-like lead compounds. These structural changes are often required, as natural products usually show unfavorable toxicities and pharmacokinetics, limiting their clinical potential (Gerry and Schreiber, 2018; Davison and Brimble, 2019). Overall, natural products have been the single most productive source of drug leads even though little of nature's biodiversity has been tested for biological activity yet (Harvey, 2000).

We therefore address and expose the general rationale for using medicinal herbs in the therapy of diseases in general and CVDs in particular. Then, we move to discuss the medicinal potentials of four traditional herbs (*Ginseng*, *Ginkgo biloba*, *Ganoderma lucidum*, and *Gynostemma pentaphyllum*) for the treatment of CVDs, which are getting increasing popularity due to their commercial commodity in many markets worldwide and to their proven therapeutic potential in several settings including cardiovascular conditions. We describe and critically discuss their therapeutic benefits in terms of molecular, cellular, and metabolic properties in the context of CVDs. In addition, we highlight the major clinical trials in which these four herbs have been used, with an emphasis on their efficacy and safety.

## Modern Medicine Management of Atherosclerosis and Hypertension

Current health care guidelines emphasize prevention to minimize the risk of CVDs (Reiner et al., 2019). This is carried out by addressing the major CVD risk factors and trying to minimize their adverse outcomes. In atherosclerosis, most therapeutic approaches aim to control hypertension and hyperlipidemia or modulate hemostasis in order to avoid thrombotic complications (Weber and Noels, 2011). Hypercholesterolemia is a major contributor in atherosclerosis, so current conventional therapeutic approaches rely significantly on lowering LDL levels using statins (Ridker et al., 2017; Aday and Ridker, 2018; Reiner et al., 2019). In cases where statin therapy does not yield a significant reduction in LDL levels, an LDL-absorption inhibitor can be used, alone or in combination with statins depending on patient response. Clinical trials have clearly shown that such therapies are effective in lowering CVD risk (Reiner et al., 2019). Recently, pro-protein convertase subtilisin/kexin type 9 (PCSK9) inhibitors were approved by the regulatory bodies as a drug that can lower LDL level, and are recommended for use in patients with heart problems, where statins were not effective at lowering LDL levels (Ridker et al., 2017; Aday and Ridker, 2018). The CANTOS clinical trial (2017) has provided evidence that in patients with elevated inflammation (hsCRP > 2 mg/L), a combination therapy of statins and canakinumab (IL-1β antibody) may be necessary to lower atherosclerosis risk (Ridker et al., 2017; Aday and Ridker, 2018; Reiner et al., 2019). Prior to the recommendations of the CANTOS study, conventional therapy regimens have neglected the role of inflammation in atherosclerosis (Weber and Noels, 2011). It is very important to highlight that complementary and alternative medicine (CAM), including herbal remedies, have already tackled the inflammatory arm of atherosclerosis much earlier than the results of the CANTOS study (Frishman et al., 2009; Orekhov et al., 2013; Al-Shehabi et al., 2016), giving a hint as to why American patients visited CAM providers much more than primary care physicians (Eisenberg et al., 1998; Tachjian et al., 2010). Of relevance to this discussion is that herbal remedies are the most common type of CAM among CVD patients (Yeh et al., 2006; Tachjian et al., 2010).

Modern therapy regimens for hypertension involve controlling BP elevations in hypertensive patients. This usually requires the use of multiple antihypertensive drug agents in the majority of these patients (Guerrero-Garcia and Rubio-Guerra, 2018). Multiple classes of antihypertensive agents are available thus offering a practitioner the ability to prescribe highly effective drug combinations in order to reduce BP and protect target organs (Stewart et al., 2019). This combination therapy is of distinctive importance in resistant hypertension, which is highly prevalent worldwide (Noubiap et al., 2019; Samaha et al., 2019).

The major drug classes available for the management of hypertension are thiazide diuretics, angiotensin-converting enzyme (ACE) inhibitors, angiotensin receptor II blockers, and calcium channel blockers (Susalit et al., 2011; Munoz-Durango et al., 2016). Vasodilators, aldosterone antagonists, β-blockers, α-blockers, renin inhibitors, and central-acting agents are other agents that are occasionally used (Omboni and Volpe, 2018). These agents lower BP in patients and reduce their risk of hypertension-related CVD events, but do not prevent them thereby justifying the use of hypertension combination therapies (Rizvi, 2017).

Despite the availability of the aforementioned medications in modern-day health care systems, high BP is managed in only 34% of the patients (August, 2004; Wang and Xiong, 2012). Such an aspect appears to be mainly related not only to the cost of antihypertensive agents (Susalit et al., 2011), but also to their availability and accessibility (Wang and Xiong, 2012), their unwanted side effects (Susalit et al., 2011; Wang and Xiong, 2012), and their low patient compliance with the required dose (August, 2004). For these factors hypertension patients seek CAM medications, especially herbal-based medicaments to treat their CVDs in general and hypertension in particular (Yeh et al., 2006; Tachjian et al., 2010; Al Disi et al., 2016).

## Herbal Medicine Management of Atherosclerosis and Hypertension

Herbal extracts and their derivatives can favorably modulate and ultimately ameliorate the molecular events that contribute to hypertension or atherosclerosis, the two major contributors to CVDs incidence. Herbal remedies contain numerous bioactives and, thus, have multi-modal cellular mechanisms of action. In fact, herbal remedies can have antioxidant, vasorelaxant, anti-inflammatory, anti-proliferative, or diuretic effects. Herbal remedies can also prevent VSMC phenotypic switching, inhibit endothelial dysfunction, platelet activation, lipid peroxidation, ROS production, and macrophage atherogenicity. Because of such a wide range of molecular and cellular targets, herbal preparations can be used to treat and manage a range of CVDs. For example, *Salvia miltiorrhiza* (Red sage), an annual sage traditionally used in Chinese medicine, has been used to treat a plethora of CVDs including CHD, myocardial infarction, atherosclerosis, and angina pectoris. The active compounds are mainly utilized as the dried root of the plant rhizome named Danshen (Gao et al., 2012). The plant bioactive compounds are the lipo-soluble Tanshinones and the water-soluble Phenolics (Ren et al., 2019). *S. miltiorrhiza* extracts have shown strong antioxidant capabilities with a high ability to scavenge free radicals, which seems the base of its strong cardio- and vascular-protective potential (Zhao et al., 2006).

Salvianolic acid B, one of the pure compounds that can be extracted from *S. miltiorrhiza*, is effective against fibrosis and ischemia–reperfusion injury (Lay et al., 2003). Danshen has a protective effect against homocysteine-induced adverse effects, where homocysteine imbalance is a high-risk factor for vascular diseases (Chan et al., 2004). In combination with *Pueraria montana* var. lobata. (Kudzu), Danshen has showed potent anti-hypertensive effects (Ng et al., 2011). In one clinical trial, Danshen capsules (1000 mg twice daily for 12 weeks) were able to significantly reduce SBP and pulse rate in patients with uncontrolled mild to moderate hypertension and under conventional antihypertensive treatment. It has also been found to be well-tolerated and considered to be safe in patients with hypertension (Yang et al., 2012).

*Astragalus membranaceus* (Synonym *Astragalus propinquus* Schischkin. in the Missouri Botanical Garden plant list), another Chinese herb, contains Astragaloside IV, which is the plant major bioactive compound widely used as an antioxidant and for protection against ischemic-associated CVDs (Zhang et al., 2006). *A. membranaceus* extract has been found to maintain cardiac functions by improving energy metabolism and inhibiting the production of free radicals in a myocardial ischemia reperfusion rat model (Zhou et al., 2000). By decreasing the levels of the oxidative stress marker malondialdehyde (MDA), maintaining superoxide dismutase (SOD) activity, and reducing free radicals-induced myocardial cell injury, *A. membranaceus* can also improve cardiac function and provide cardioprotection in a myocardial ischemic rat model (Ma et al., 2013). *A. membranaceus* extract also has angiogenic effects in the ischemic injury rat model (Zhang et al., 2011). Astragaloside IV has been found to provide a positive inotropic effect improving left ventricular ejection in patients with congestive heart failure (CHF) (Luo et al., 1995). The polysaccharide of *A. membranaceus* has also been shown to reduce insulin resistance and to possess anti-obesity and hypolipidemic effects (Mao et al., 2009).

*Allium sativum* (Garlic) is a classic example of herbs used in CVDs management and is quite known for its multifaceted properties against CVD-associated conditions such as hypertension, oxidative stress, inflammation, and hyperlipidemia (Ashraf et al., 2013; Jeong et al., 2016; Thomson et al., 2016). Indeed, by reducing total cholesterol and LDL levels, decreasing the content of lipid in arterial cells and inhibiting VSMCs proliferation, garlic can be used to manage atherosclerosis and hyperlipidemia (Sun Y. et al., 2018). Owing to its endothelial NO synthase (eNOS)-modulated vasorelaxation ability, *Crataegus oxyacantha* (Synonym *Crataegus rhipidophylla Gand*. Common name Hawthorn) is another example of herbs commonly used to manage hypertension (Brixius et al., 2006). Another herb, *Crocus sativus* (Saffron), can block Ca^2+^ channels *via* endothelium-independent mechanisms providing another vasodilator mechanism, in addition to its eNOS activating ability (Razavi et al., 2016). Among other medicinal plants *Hibiscus sabdariffa* (roselle), is known to reduce BP using its ability to inhibit ACE (Ojeda et al., 2010), while *Camellia sinensis* (Tea) extracts can reduce hypertension by significantly increasing brachial artery flow-mediated dilation (FMD) (Ras et al., 2011). Rosemary (*Rosmarinus officinalis*) exhibits neuroprotection by acting against ischemic stroke-associated cerebral insufficiency, which is characterized by a reduction of localized blood flow in the brain. Through its anti-inflammatory properties, rosemary can decrease the expression of inducible NO synthase (iNOS) and cyclooxygenase-2 (COX-2) as well as that other pro-inﬂammatory enzymes and mediators (Seyedemadi et al., 2016). The use of herbal plants extends to include CHF and atrial arrhythmias. Digitalis, extracted from the dried leaves of the common foxglove, is a potent inhibitor of Na^+^/K^+^-ATPase and can cause depolarization leading to smooth muscle contraction and vasoconstriction and hence can strengthen muscle heart contractions (Liu et al., 2016).

Given all these restorative abilities, it is not surprising that herbal remedies are being absorbed into evidence-based medicine for the prevention and/or treatment of CVDs. **Table 1** lists common herbal remedies and the form of CVDs that they can help alleviate.

**Table 1:** Some herbal remedies traditionally used for the treatment of different forms of CVDs.

CVD form	Examples of herbal remedies used
Atherosclerosis and hyperlipidemia	Garlic (*Allium sativum*)
	*Commiphora mukul*
	*Monascus purpureus*
	Berberine (active compound of *Coptis chinensis*)
Systolic hypertension	Garlic (*Allium sativum*)
	*Rauvolfia serpentina*
	*Panax* species (Ginseng)
	*Stephania tetrandra*
	*Veratrum* species alkaloids
	*Ligusticum wallichii* (Synonym of *Ligusticum striatum* DC)
	Hawthorn from *Crataegus oxyacantha*
	*Camelia sinensis*
	*Andrographis paniculata*
	*Apium graveolens*
	*Bidens pilosa* L.
	*Crocus sativus*
	*Cymbopogon citratus*
	*Hibiscus sabdariffa*
	*Nigella sativa*
	*Urtica dioica*
	*Viola odorata*
	*Mentha longifolia*
	*Salvia miltiorrhiza*
	*Uncaria Rhynchophylla*
Venous insufficiency	*Aesculus hippocastanum* *Ruscus aculeatus*
Cerebral insufficiency	*Ginkgo biloba* *Rosmarinus officinalis*
Angina pectoris	*Crataegus species*
	*Panax notoginseng*
	*Salvia miltiorrhiza*
Congestive heart failure	*Digitalis purpurea*
	*Digitalis lanata*
	*Crataegus species*
	*Adonis microcarpa* and *Adonis vernalis*
	Berberine (active compound of *Coptis chinensis*)
	*Salvia miltiorrhiza*

The herbal remedies refer to the plant extract, unless otherwise indicated where the remedy may be a purified or partially purified active ingredient of the plant extract. Data were obtained from published data in (Valli and Giardina, 2002; Frishman et al., 2009; Al Disi et al., 2016; Al-Shehabi et al., 2016; Samaha et al., 2019).

Although herbs have been widely used in both traditional and modern medicine, a limited number of reviews that gather them and comprehensively focus on their mechanisms of action and safety in the context of CVDs are present. Many plant-based compounds appear to have cardiovascular protective effects, nevertheless, among the most effective compounds are flavonoids, terpenoids, saponins, and polysaccharides. These highly effective compounds are major components of four of the most recognized herbal preparations namely: Ginseng, *Ginkgo biloba*, *Ganoderma lucidum*, and *Gynostemma pentaphyllum*, which we decide to cover in this review.

## Ginseng

**Figure 2:**
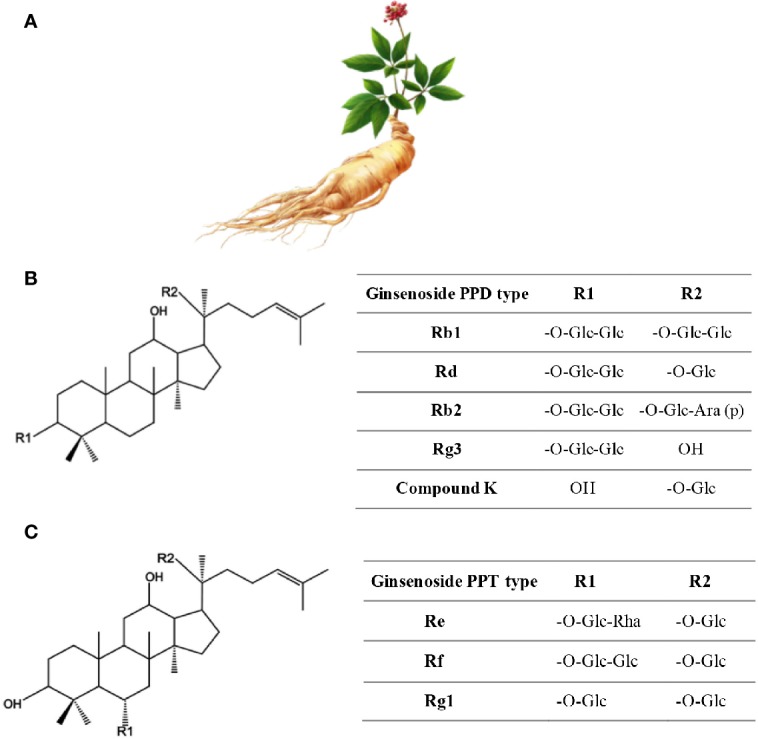
Ginseng. **(A)** Roots have the healing properties (from https://pngtree.com/freepng). **(B)** Chemical structure of Ginsenosides. **(C)** Ginsenosides protopanaxadiol (PPD) and Ginsenoside protopanaxatriol (PPT). R1 and R2 are side chains in different ginsenosides. Glc, glucose; Ara, arabinose; Rha, rhamnose.

Ginseng is an anciently cultivated plant (2000 years ago) partly due to its ritual use (**Figure 2**). Ginseng use in traditional medicine goes back to 20 centuries ago (Kim, 2012), but its use in Western medicine dates back to the early 20^th^ century by two British physicians F. Porter Smith and G.A. Stuart who were exploring Chinese herbal remedies at the time (Shih-Chen et al., 1973). Ginseng habitats include Asian countries such as Korea, China, Japan, and Vietnam, and North American countries, mainly Canada and the United States. Korean red ginseng (KRG; *Panax ginseng C.A. Mey*.), Chinese ginseng (*Panax notoginseng* Burkill; F.H.Chen.), American ginseng (*Panax quinquefolium L*.), and Japanese ginseng (*Panax japonicas* C.A. Mey.) represent the most commonly used ginsengs.

Usually the roots of 5 to 7-year-old plants are either air-dried under the sun yielding “white ginseng” or steam-treated at 98–100°C for 2–3 h and then sun-dried to produce the “red ginseng” (Kim et al., 2000). During steaming, ginseng chemical constituents undergo changes that make red ginseng more pharmacologically effective than white ginseng (Kim et al., 2000). Currently, ginseng is prepared and used either in a liquid form: oil extracts or tea; or in a solid form: tablets, capsules, or dried roots (Valli and Giardina, 2002). However, extracts of ginseng root, berry, and leaf have been repeatedly demonstrated to have anti-obesity, anti-hyperglycemic, anti-hypertensive, insulin sensitization, and anti-hyperlipidemic effects (Kim, 2012).

More than 300 bioactives have been isolated from Ginseng. Ginsenosides, which are triterpene saponins, are the most bioactive constituents isolated from Ginseng extracts (Mahady et al., 2000). Of the 40 ginsenosides isolated so far, Rb1, Rg1, Rg3, Re, and Rd are the most frequently studied. Rg3, Rg5, and RK1 are unique to the red Korean Ginseng (**Figure 2**) (Lee and Kim, 2014). Research into Ginseng and its constituents has flourished so that currently there is a journal dedicated to Ginseng research: Journal of Ginseng Research (https://www.journals.elsevier.com/journal-of-ginseng-research). The study of the purified individual ginsenosides rather than the whole Ginseng root extract has gained recent interest (Kim, 2012). Ginseng and its ginsenoside constituents have vasorelaxation, anti-oxidation, anti-inflammation, and anti-cancer activities (Kim, 2012; Choi J. et al., 2013).

### Ginseng at the Bench: Mechanism of Action in CVDs

In the context of CVDs, Ginseng has been used to manage hypertension. Ginseng has hypotensive effects due to its effect in the improvement of arterial functions. Interestingly, ginsenosides facilitate vasorelaxation of different vessels: rat aortas (Kim et al., 1999a), murine coronary arteries (Pan et al., 2013), and monkey cerebral arteries (Toda et al., 2001). Ginseng can increase eNOS expression and NO production while ginsenoside Rg3 activates eNOS (Valli and Giardina, 2002; Jang et al., 2011; Hong et al., 2012; Pan et al., 2013; Lee K. et al., 2016). KRG induces NO-dependent vasorelaxation improving vascular tone. These effects are mediated by the inhibition of arginase activity, the increase of NO generation, and the enhancement of eNOS dimer formation (Shin W. et al., 2013). The *Panax ginseng* G115 extract has also been shown to inhibit ACE activity in human umbilical vein endothelial cells (HUVECs) and angiotensin I-induced contractions of bovine mesenteric arteries (Persson et al., 2006). Other Ginseng CVDs management properties are its anti-oxidant (Lee et al., 2019a), anti-inflammatory (Keum et al., 2003; Shin Y. et al., 2013), and anti-hyperlipidemic (Park et al., 2005) effects, along with its ability to regulate Ca^2+^ channels (Lee and Kim, 2014).

The ginsenoside Rg3 can increase NO and cGMP levels, activate Ca^2+^-gated potassium channels, inhibit ACE activity, and block Ca^2+^-gated channels (Kim et al., 1999b; Persson et al., 2006; Park J. et al., 2014). Ginseng has also demonstrated an anti-inflammatory role by inhibiting the activation of activator protein (AP-1) and nuclear factor-kappa B (NF‐κB), ultimately reducing the expression of COX-2, IL‐6, IL‐1β, and tumor necrosis factor‐α (TNF‐α) (Keum et al., 2003; Shin Y. et al., 2013). In macrophages, Baek et al. demonstrated that each fraction of the KRG exerts anti-inflammatory actions through a different mechanism. For instance, the saponin fraction significantly suppressed NO production and reduced the expression of inflammatory genes such as iNOS, COX-2, TNF-α, and interferon-β. In contrast, all extracts, including water extracts, saponin, and non-saponin fractions, inhibit the activity of the kinase TBK1 and suppress both nuclear translocation and transcriptional activity of its downstream effector interferon regulatory factor 3 (IRF3) (Baek et al., 2016).

By inhibiting diacylglycerol liberation, dietary supplementation of KRG lowers blood cholesterol levels and reduces the formation of atherosclerotic lesions induced by a high cholesterol diet in rabbit (Hwang et al., 2008). Again, by up-regulating the adenosine triphosphate-binding cassette transporter A1, *the* saponin fraction of *P. notoginseng* can attenuate cholesterol esters in foam cells (Jia et al., 2010). In addition, Ginseng has shown a potent *in vivo* antithrombotic effect, which may be due to an antiplatelet activity rather than an anticoagulation activity, indicating that Ginseng intake may be beneficial for individuals with high risks of thrombosis and CVDs (Lee and Kim, 2014). In this context, the dihydro-ginsenoside Rg3 has been reported to potently inhibit platelet aggregation through the modulation of downstream intracellular signals such as cAMP and extracellular signal-regulated kinase 2 (Lee et al., 2008).

### *In Vivo* Preclinical Evaluation of Ginseng in Animal Models of CVDs

Ginseng's hypotensive effects have been extensively demonstrated (Valli and Giardina, 2002; Lee et al., 2012; Mucalo et al., 2013; Al Disi et al., 2016). For example, Ginseng can reduce adrenal catecholamines secretion in hypertensive rats, thus contributing to vasorelaxation (Jang et al., 2011). However, there are reports of Ginseng being hypertensive (Jang et al., 2011; Kim, 2012). In fact, Ginseng may have biphasic concentration-dependent effects. Low doses of ginseng raise BP, while higher concentrations repress BP (Jang et al., 2011), a phenomenon that could be due to the varied action of different Ginseng extract constituents (Valli and Giardina, 2002).

Through their antioxidant properties, ginsenosides also mediate anti-hypertensive and anti-atherosclerotic effects. Ginsenosides exhibit free radical scavenging and metal ion chelating abilities. For instance, the lipid-soluble and water-soluble extracts from the North American Ginseng exhibit strong antioxidant activity (Kitts et al., 2000). Lu et al. showed that Rb1 could significantly and specifically alleviate hydroxyl radical and hypochlorous acid radical damaging effects (Lu et al., 2012). Aged rats, supplemented with the North American Ginseng for four months, had decreased production of both ROS and age-related oxidative damage in proteins of the heart and muscle fibers, a phenomenon mediated by SOD and glutathione peroxidase (GSH-Px) activation (Fu and Ji, 2003). The ginsenoside Rg3 can ameliorate mitochondrial dysfunction and promote enhanced expression of antioxidant proteins, such as the nuclear factor erythroid 2-related factor-2 (Nrf2) and the heme oxygenase-1 (HO-1) (Lee et al., 2019a). By reducing hypertension- and atherosclerosis-associated inflammatory states, Ginseng can reduce CVD risk. To address the anti-inflammatory effects of Ginseng, Mohammadi et al. carried out a meta-analysis of data from randomized controlled trials. They report that Ginseng supplementation significantly lowered the levels of two key pro-inflammatory mediators IL‐6 and TNF‐α (Mohammadi et al., 2019). By modulating angiogenesis (decreased VEGF-A and FGF-2 expression), inflammatory (decreased CD68, TNFα, and MCP-1 expression) and matrix metalloproteinase (MMP) activity, Ginseng can inhibit ovariectomy-induced obesity, adiposity, and adipocyte hypertrophy (Lee H. et al., 2016).

Ginseng can lower the risk of atherosclerosis by inducing a better lipid profile. Ginseng's beneficial effects on lipid metabolism have been described more than three decades ago (Qureshi et al., 1983; Yamamoto et al., 1983). In humans and rats, red Ginseng supplementation improves lipid profiles by diminishing the total plasma levels of cholesterol, triglycerides, LDL-C, free fatty acids (FFA), and platelet adhesiveness and increasing HDL-C levels in total plasma (Deng et al., 2017; Singh et al., 2017). In rats, black Ginseng can ameliorate hypercholesterolemia by interfering with the expression of cholesterol metabolism genes (Saba et al., 2016). In addition, lipid profiles tend to improve in diabetic rats receiving Ginseng, suggesting that *Panax ginseng* can ameliorate diabetes mellitus-initiated dyslipidemias (Deng et al., 2017; Abdelazim et al., 2018). By modulating the secretion of lipoproteins, Ginseng can reduce the microsomal triglyceride transfer protein (MTTP) (Oh et al., 2012), which plays an essential role in lipid metabolism and transport (Deng et al., 2017).

KRG works as an agonist of peroxisome proliferator-activated receptor (PPAR), which is known to improve atherogenic dyslipidemia by augmenting liver PPAR-α mRNA and raising lipoprotein lipase mRNA levels (Park et al., 2005). Consistent with this study, Shin et al. demonstrated that Ginseng could prevent obesity and dyslipidemia in high-fat diet (HFD)-fed castrated mice. These processes were mediated through the inhibition of adipogenesis-related genes expression (SREBP-1C, PPAR-γ, FAS, SCD1, and ACC1) in visceral adipose tissues (Shin and Yoon, 2018). Ginseng extracts or ginsenosides can act synergistically with testosterone to further inhibit dyslipidemia (Shin and Yoon, 2018). Mollah et al. also showed that Ginseng can improve lipid profiles *via* PPAR pathway activation (Mollah et al., 2009; Yang and Kim, 2015). Further, ginsenoside Rg1 can activate the promoter of PPAR-α leading to the expression of its target genes carnitine palmitoyltransferase-1 (CPT-1) and acyl-CoA oxidase (ACO), which are involved in fatty acid oxidation. These findings indicate that Rg1-induced improvement of lipid profiles may be associated with increased fatty acid oxidation through PPAR-α activation (Park et al., 2011).

### Ginseng to the Clinic

Numerous clinical trials have been conducted to assess the cardio-protective and beneficial effects of Ginseng and its constituents in CVD treatment. Checking the clinical trials on Ginseng, its extracts or ginsenosides on the U.S. National Library of Medicine www.clinicaltrials.gov reveals that there are 162 Ginseng clinical trials. Of the 162 trials, 47 were Phase 3 or 4 trials, 97 have been completed and the rest are ongoing. Importantly, a significant number of these trials addresses CVDs. For example, eight trials addressed hypertension, five addressed arterial occlusive diseases, and another five addressed strokes. One such clinical trial examined the vasorelaxation effects of Asian Ginseng (AG) and its ability to modulate vascular function. Trial participants were randomized to either the selected AG extract or placebo groups and received a daily dose of 3 g of AG for 12 weeks in combination with their usual antihypertensive and anti-diabetic therapy. Combining AG extract with conventional therapy in diabetics patients with concomitant hypertension decreased arterial stiffness and attenuated SBP (Mucalo et al., 2013). Another clinical trial found that Rg3 from KRG lowers central and peripheral arterial pressures in healthy adults (Jovanovski et al., 2014). In an acute, randomized, placebo-controlled, double-blind, crossover trial on participants with type 2 diabetes mellitus (T2DM), Shishtar et al. showed that acute administration of Korean white Ginseng appears to be safe and exhibits beneficial effects on the augmentations index, a cumulative indicator of arterial health (Shishtar et al., 2014a).

A 12-week intervention with KRG was conducted in patients with impaired fasting glucose, impaired glucose tolerance, or newly diagnosed with T2DM. Subjects were randomized in a double-blind, placebo-controlled trial. The trial results showed that 12 weeks of intervention with KRG supplementation (5 g/day) led to normalization of whole blood and serum glucose levels as well as serum insulin and CRP concentrations (Bang et al., 2014). Administration of *Panax Ginseng* extract (PGE) for 8 weeks (6 g/day) decreased serum triglycerides and total cholesterol and LDL levels, while increasing HDL levels. These results were attributed to PGE potent antioxidant effects (Kim and Park, 2003). In accordance, the effects of a low-dose (3 g/day) and a high-dose (6 g/day) of KRG supplementation for 8 weeks on antioxidant enzymes and oxidative stress markers in humans were assessed in a randomized, double-blind, placebo-controlled trial. Increased GSH-Px, SOD, and CAT activities were found in the high-dose group as compared to the placebo group. Plasma oxidized-LDL levels and DNA tail length and tail moment were significantly decreased in both high and low dose groups but increased in the placebo group. This led to the conclusion that supplementation with KRG upregulates antioxidant enzymes activities and consequently attenuates lymphocyte DNA damage (Lee et al., 2012).

The efficacy of Ginseng against T2DM is well documented. A meta-analysis of eight clinical trials showed that administration of Ginseng, in comparison to the placebo, improves fasting glucose levels, postprandial insulin levels, and insulin resistance. In these patients, ginseng was able to improve blood lipid profile lowering triglycerides, total cholesterol, and LDL levels. This meta-analysis concluded that Ginseng supplementation can improve the control of glucose levels and insulin sensitivity in patients with T2DM (Gui et al., 2016). Another meta-analysis of 16 randomized clinical trials was conducted to assess the efficacy of Ginseng in controlling glycemic index by reporting the ability of Ginseng to reduce fasting blood glucose in both patients with and without diabetes (Shishtar et al., 2014a; Shishtar et al., 2014b). Interestingly, when Ginseng is combined with conventional drugs, its efficacy in the management of hypertension is more pronounced. Indeed, combining AG extract with conventional therapy in diabetic patients with concomitant hypertension decreased arterial stiffness and attenuated SBP (Mucalo et al., 2013).

Despite the numerous clinical trials showing the efficacy of Ginseng in CVDs management, this aspect yet remains controversial. In fact, some studies could not demonstrate Ginseng's beneficial effects against CVD. For example, a meta-analysis of 17 randomized clinical trials (1381 participants) found no significant effect of AG on arterial BP and hence no effect on CVDs risk (Komishon et al., 2016). Another clinical trial concluded that KRG intake (3 g/day) for 3 weeks had no beneficial effects on arterial stiffness in subjects with hypertension (Rhee et al., 2011). Yet, as mentioned above, when KRG was combined with conventional therapy it was able to control hypertension (Rhee et al., 2011).

Thus, Ginseng appears to be efficient in regulating several lipid profile parameters, and has shown positive effects in patients with T2D. Also, the efficacy of Ginseng in the management of hypertension is well documented when combined with conventional hypotensive medications.

### Safety, Toxicity, and Side Effects of Ginseng

As mentioned earlier, the claimed safety of medicinal herbs has to be handled cautiously, and on a case-by-case basis for each herbal preparation. The safety of Ginseng has been experimentally approached using animal models and human clinical studies (Mahady et al., 2000). An abundant number of *in vitro* and *in vivo* studies, as well as human clinical trials have pointed out that Ginseng extracts have negligible side effects (Park K. et al., 2014). Few unfavorable symptoms were reported following long periods of administration of high doses of Ginseng extracts. This included morning diarrhea, skin eruption, nervousness, sleeplessness, hypertension, edema, decreased appetite, depression, and hypotension (Siegel, 1979; Kiefer and Pantuso, 2003). A systematic review on PGEs in randomized controlled trials highlighted the safety of Ginseng. The review identified 40 studies where adverse effects were reported, but analysis revealed that out of the 40 studies, 16 studies showed no adverse events and 24 studies had 135 minor events (Shergis et al., 2013). Lee et al. reported that *P. ginseng* extract (1 or 2 g/day) supplemented over the course of 4-weeks was safe, tolerable, and free of toxic effects in healthy volunteer subjects. Only non-significant changes were observed in hematological and biochemical tests (Lee et al., 2012). Recently, Song et al. performed a large-scale clinical study with 1000 participants randomly divided into two groups; a placebo and a group supplemented with 2 g/d of KRG. Their findings asserted the safety and tolerability of KRG (Song et al., 2018).

Along the same lines, the mutagenic and toxicity potentials of tissue cultured mountain Ginseng adventitious roots (TCMGARs) were tested. TCMGARs did not exhibit any mutagenic properties when tested in diverse strains of *Salmonella typhimurium* and *Escherichia coli*. This was further shown *in vivo* without any evidence of TCMGARs mutagenicity, such as chromosomal aberration and micronucleus appearance, in mice exposed to TCMGARs (Murthy et al., 2018). All these studies confirm the biosafety and non-toxicity of Ginseng at an average dietary consumption.

Ginseng supplements have also shown certain clinically relevant patterns of adverse cardiovascular reactions. There are reports of numerous cases where prolonged Ginseng use or misuse has led to potential side effects related to cardiovascular events such as increased BP (Coon and Ernst, 2002), long QT syndrome, or atrial fibrillation (AF) (Paik and Lee, 2015). For example, in a young man, 3-year Ginseng supplementation has been found to correlate with hypertension, shortness of breath, dizziness, and inability to concentrate, symptomatology that disappeared and did not recur after stopping the supplements. In another instance, a hypertensive female receiving no other medication than Ginseng (Ginzin tablets; Ferrosan) reported an increase in BP rather than a decrease. Interestingly, such Ginseng-associated BP increase remitted going back to pre-treatment levels 4 days after the cessation of Ginseng intake (Coon and Ernst, 2002). Although the observed effects appeared not to be clinically relevant, in a 30-subjects prospective, randomized, double-blind, placebo-controlled study, Ginseng was found to prolong the QTc interval and reduce DSB in healthy adults as early as 2 h after consumption (Caron et al., 2002). A 43-year-old healthy woman without familial history of sudden cardiac death and negative test of long QT mutations developed a long QT syndrome followed by polymorphic ventricular tachycardia. The woman admitted to the hospital revealed she was consuming 70 cL of caffeine and 4 L of Korean *Panax ginseng* daily for 6 months. Upon stopping Ginseng consumption, the patient had no subsequent events. Yet, it is not proven whether a higher dose of Ginseng or a synergistic effect of caffeine could further prolong QT leading to malignant dysrhythmias (Torbey et al., 2011). Additionally, an AF with slow ventricular rate developed after taking AG for 1-week in an 83-year old woman with chronic renal disease (Liao et al., 2010). Nevertheless, all these mentioned episodes are considered rare adverse reactions that mostly depend on inter-variability between patients (Paik and Lee, 2015).

Ginseng has been reported to interact with several drugs, yet its interaction with warfarin (blood-thinning drug) is the most documented (Yuan et al., 2004; Chua et al., 2015). A randomized, double-blind, placebo-controlled trial using 20 healthy patients concluded that a 2-week intake of American Ginseng (2 g/d; 1 g twice daily) significantly reduced peak international normalized ratio (INR) and peak plasma warfarin levels (Yuan et al., 2004). In a recent study performed on rats, ginsenosides were reported to significantly enhance the activity of two enzymes known to metabolize warfarin, P450 CYP3A4 and P450 CYP2C9, restoring the levels of coagulation factors II and VII and that of the protein Z, that are usually suppressed by warfarin (Dong et al., 2017). The combined use of *Panax ginseng* with the monoamine oxidase inhibitor, phenelzine (Nardil), may result in manic-like symptoms (Vogler et al., 1999). Finally, although the efficacy and safety of Ginseng has been evidenced in numerous clinical studies, additional well-designed, large-scale randomized control trials are needed.

## Ginkgo biloba

**Figure 3:**
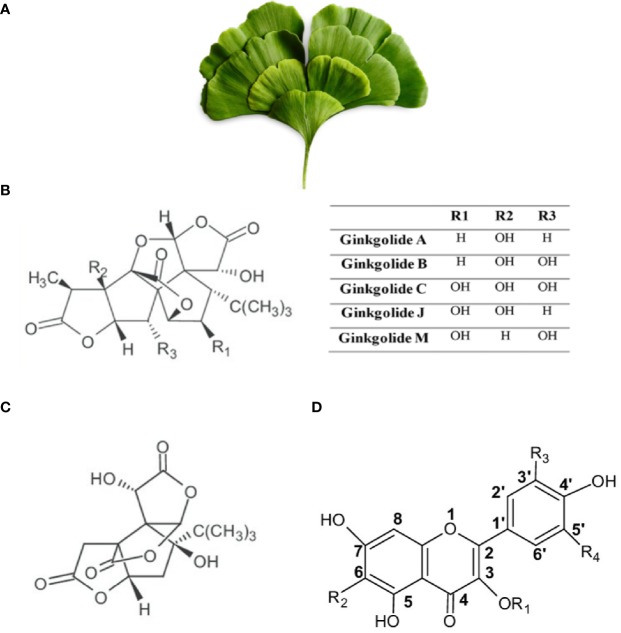
*Ginkgo biloba*. **(A)** Leaves of *Ginkgo biloba* or Maiden Hair Tree (from https://pngtree.com/freepng). **(B)** Chemical structure of Ginkgolides. **(C)** Chemical structure of Bilobalides. **(D)** Structural skeleton of flavonoids. R1 and R2 are side chains.

*Ginkgo biloba*, also known as the maidenhair tree in English due to its resemblance to the foliage of the Maidenhair fern (**Figure 3**), is among the oldest seed plants. It is regarded as a “living fossil” because of its continued existence without dramatic changes for 270 million years. (Hori et al., 1997). Its place of origin is believed to be eastern China in Yangtze River Valley (Jaggy and Koch, 1997; Singh et al., 2008). From there, it became extensively distributed in Asia, Europe, North America, and New Zealand and is now widely cultivated (Kleijnen and Knipschild, 1992; Hori et al., 1997; Belwal et al., 2019). A remark about its leaf extract is included in the medical Dictionary of the Republic of China (Kimbel, 1992; Kleijnen and Knipschild, 1992; Kressmann et al., 2002). *Ginkgo biloba* is the only living species of the division Ginkgophyta probably due to its resistance to environmental stresses (Deng et al., 2006; Cao et al., 2012).

*Ginkgo biloba* is one of the most sold medicinal plants. It is one of the herbs mentioned in the Chinese Materia Medica more than 5000 years ago, where its seeds and leaves—fresh or dried—have been used for thousands of years in ancient herbal medicine. Current research on its therapeutic properties mainly uses *Ginkgo biloba* leaves and many pharmaceutical companies including those in the USA and Europe manufacture and sell extracts of the leaves (Kimbel, 1992; Kleijnen and Knipschild, 1992; Kressmann et al., 2002). The leaves can be used for the treatment of asthma and bronchitis in the form of a tea that is most commonly used by the Chinese people. More commonly, a standardized extract containing the most active constituents can be made from the leaves and then taken as a tablet, in liquid form, or given intravenously (Kleijnen and Knipschild, 1992).

The main constituents of *Gingko biloba* are flavonoids (ginkgo-flavone glycosides), terpenoids (ginkgolides and bilobalides), biflavones, and organic acids among other substances (**Figure 3**). Ginkgolides, being unique to *Gingko biloba*, are not synthesized by any other living species. Ginkgolides are classified into either A, B, C, J, or M types (**Figure 3**). *Gingko biloba* flavonoids include several representative glycosides, such as kaempferol, quercetin, and isorhamnetin (**Figure 3**). Flavonoids are known to reduce free radical generation and terpenoids are known to reduce inflammation and protect nerve cells against neuro-inflammation (Kleijnen and Knipschild, 1992; Ude et al., 2013; Isah, 2015). Through a multistep process, *Ginkgo biloba* dried leaves extracts are enriched for flavonoids and terpenoids and the unwanted substances are eliminated. At the final step, the liquid extract is dried to give 1 part extract from 50 parts of raw drug (leaves) (Kleijnen and Knipschild, 1992; Isah, 2015). The composition of *Gingko biloba* extracts may differ depending on the manufacturing process. Standardized extract forms have been developed and usually contain 24–36% flavone glycosides and 4–11% terpenoids. For example, standardized extract EGb761 is the most commonly used *Ginkgo biloba* extract (GBE), and it contains 24% ginkgo flavonoid glycosides, 6% terpene lactones, and 5–10% organic acids (Kressmann et al., 2002; Chan et al., 2007). These extracts have been used for various therapeutic purposes, including regulation of cerebral blood flow (Mashayekh et al., 2011), protection against free radicals (Oyama et al., 1996; Bridi et al., 2001), tinnitus treatment (Mahmoudian-Sani et al., 2017), protection of neurons (Mahdy et al., 2011), as well as enhancement of cognitive functions, such as memory and concentration problems (Weinmann et al., 2010; Tan et al., 2015).

### *Ginkgo biloba* at the Bench: Mechanism of Action in CVDs

*Ginkgo biloba's* therapeutic effects and pharmacological actions are majorly due to its constituent flavonoids (ginkgo-flavone glycosides) and terpenoids (ginkgolides and bilobalide) (Lacour et al., 1991). These *Ginkgo biloba* constituents are well known for their antioxidant and anti-inflammatory effects. *Ginkgo biloba* antioxidant and anti-inflammatory effects are beneficial in a plethora of diseases that include cardiovascular, pulmonary, and central nervous systems.

Free radical generation contributes to the development and progression of numerous CVDs, including vascular injuries and atherosclerotic plaque formation. During CVD pathogenesis, the equilibrium between free radical generation and antioxidant defense is greatly shifted toward the former (Singh and Niaz, 1996; Witztum and Berliner, 1998; Fulton and Barman, 2016). GBE greatly restores the disturbed oxidative state equilibrium due to their antioxidant action, which helps to scavenge excessive free radicals as well as reduce free radical generation.

In addition, vasodilatory and antihypertensive properties of GBE can exert cardioprotective benefits (Perez-Vizcaino et al., 2009). In this regard, GBE has exhibited ACE inhibitory activities (Mansour et al., 2011), activation of cholinergic pathways, endothelial health improvement, inhibition of endothelium activation and adhesion (Mesquita et al., 2017), and serum lipid-lowering activities (Liou et al., 2015; Huang et al., 2018) among other reported effects that are beneficial in CVD.

By acting as an anti-atherothrombotic and anti-inflammatory agent, GBE can limit LPS-induced proliferation of VSMCs and their morphological alterations. Furthermore, GBE can regulate the inflammatory response in blood vessels by decreasing the activity of the ROS producing enzyme, nicotinamide adenine dinucleotide phosphate (NADPH) oxidase (NOX), and reducing the phosphorylation of mitogen-activated protein kinases (MAPKs). Subsequently, MAPKs suppress toll-like-receptor-4 (TLR-4) expression in human aortic smooth muscle cells (Lin et al., 2007). GBE can also decrease the production of the enzyme involved in the rupture of atherosclerotic plaques, MMP-1, in oxidized LDL- and 4-hydroxynonenal-induced human coronary smooth muscle cells (Akiba et al., 2007). In the same model, the GBE constituent, Ginkgolide B, attenuated endothelial dysfunction by inhibiting monocyte chemotactic protein‐1 (MCP‐1), intercellular adhesion molecule‐1 (ICAM‐1), and vascular cell adhesion molecule‐1 (VCAM‐1) production in oxidized‐LDL‐induced HUVECs. Additionally, Ginkgolide B treatment reduced the expression of several inflammatory cytokines in oxidized‐LDL‐induced mouse RAW264.7 macrophages (Feng et al., 2018). Ginkgolide C, another GBE constituent, can reduce adipogenesis and enhance lipolysis leading to suppression of lipid accumulation. Ginkgolide C treatment of 3T3-L1 adipocytes decreased the expression of PPAR adipogenesis-related transcription factors. Ginkgolide C also enhanced the Sirt1/AMPK pathway resulting in decreased activity of acetyl-CoA carboxylase and fatty acid synthesis. Moreover, Ginkgolide C stimulated the production of adipose triglyceride lipase and hormone-sensitive lipase, leading to elevated lipolysis levels (Liou et al., 2015). Similar results were obtained with human HepG2 hepatocyte cell line (Huang et al., 2018).

### *In Vivo* Preclinical Evaluation of *Ginkgo biloba*

*Ginkgo biloba* has several cardioprotective effects, including improvement of atherosclerosis due to their ability to block platelet-activating factor and platelet aggregation in rats (Zeng et al., 2013; Huang et al., 2014).

eNOS is responsible for most of the vascular NO production, and NO acts as a protective molecule to maintain vasculature hemostasis and protection of the vascular endothelium (Forstermann and Munzel, 2006). eNOS production and activity are impaired in several CVDs, including hypertension (Chou et al., 1998), cardiac hypertrophy (Ozaki et al., 2002), myocardial infarction (Tsutsui et al., 2008), and heart failure (Couto et al., 2015). GBE can act as an antihypertrophic agent by the activation of the M_2_ muscarinic receptors/NO pathway and of cholinergic signaling during cardiac hypertrophy. In a rat model of chronic β-adrenergic stimulation-induced cardiac hypertrophy, GBE was able to ameliorate the deleterious cardiac events associated with cardiac hypertrophy. These effects were mediated by the upregulation of M_2_ receptors and the downregulation of β_1_-adrenergic receptors. GBE also restored eNOS activity and consequently elevated NO levels (Mesquita et al., 2017). In addition, the anti-hypertensive effects of EGb761 supplementation were documented in hypertensive rats where SBP, DBP, and arterial BP were reduced. EGb761 supplementation also decreased inflammation and oxidative stress. While eNOS protein expression levels were enhanced, protein levels of iNOS were decreased (Abdel-Zaher et al., 2018).

Vascular aging is commonly accompanied with low-grade inflammation and degenerative structural changes and stiffness of blood vessels and is considered a risk factor for the development of CVDs, such as CHD and hypertension (Franceschi et al., 2000; Lakatta and Levy, 2003). In the mesenteric arterioles of old rats, GBE had a protective effect that alleviated arterial stiffness and improved endothelial health (Cuong et al., 2019). In these aged mesenteric arterioles, GBE improved vascular elasticity by narrowing the EC gap, increasing curvature of inner elastic membrane and reducing the middle collagen fiber layer. These changes were accompanied by decreased phosphorylation levels of Akt/FoxO3a signaling components, which usually contributes to vascular dysfunction (Cuong et al., 2019).

Pre-treatment with EGb761 in rats that have undergone myocardial ischemia–reperfusion injury inhibited the apoptosis of myocardial cells, decreased the expression of caspase 3 and pro-apoptotic Bax and increased that of anti-apoptotic Bcl-2, and protected the myocardium by activating the endogenous Akt/Nrf2 antioxidant stress pathway. Akt/Nrf2 activation subsequently decreased oxidative stress leading to reduced lipid peroxidation and increased activities of the endogenous anti-oxidant defense enzymes, namely SOD, and GSH-Px. In addition, EGb761 pre-treatment increased the expression of the heat shock protein heme oxygenase 1 (HO-1) and repressed the expression of mediators of the inflammatory response, such as TNF-α, IL-6, and IL-1β (Chen et al., 2019). HO-1 degrades heme (a potent oxidant) to generate carbon monoxide, which has anti-inflammatory properties, bilirubin, which is an antioxidant derived from biliverdin, and iron (He et al., 2014). Similar *Ginkgo biloba* anti-oxidant properties have been reported in diabetic rats as well. Administration of GBE for 30 days can increase SOD, CAT, and GSH-Px activity along with glutathione (GSH) levels in the liver and pancreas of diabetic rats (Cheng et al., 2013). This enhanced anti-oxidant status might be responsible for improved glucose uptake *via* increased GLUT-4 expression (Shi et al., 2010). Furthermore, EGb761 oral supplementation of HFD-fed mice can dose-dependently enhance glucose tolerance, decrease insulin levels, and diminish parameters of insulin resistance (Cong et al., 2011).

The above reports point that GBE has a pleiotropic mechanism of action. Indeed, a metabolomic profiling study of the plasma and hearts of GBE-supplemented rats with myocardial infarction established that GBE acts *via* the regulation of multiple metabolic pathways. Metabolomic profiles of rats with MI showed disturbed metabolism in these rats because of modulated inflammatory reaction, oxidative stress, and structurally damaged pathways. However, GBE supplementation controlled the inflammatory reaction and oxidative stress pathways by regulating sphingolipid, phospholipid and glyceride metabolism and ameliorated the structural damage by downregulating amino acid metabolism (downregulation of urea cycle) and decreasing oxidative stress (Wang et al., 2016).

In addition to the above-mentioned effects, GBE was able to decrease calcium overload (Liu et al., 2013), the primary factor responsible for the irreversible myocardial injury (Moens et al., 2005). Rats with an ischemic myocardium and pre-treated with GBE50, an extract that matches EGb761, exhibited decreased intracellular calcium overload which could block arrhythmia. GBE could decrease the calcium overload and protect from an ischemic myocardium by inhibiting the Na^+^/Ca^2+^ exchanger (Liu et al., 2013).

### *Ginkgo biloba* to the Clinic

Given the above reported protective and therapeutic benefits of GBE *in vitro* and *in vivo*, several clinical trials have been conducted to test different formulations and doses of GBE in a plethora of diseases (DeKosky et al., 2008; Gardner et al., 2008; Kuller et al., 2010; Hashiguchi et al., 2015). A search of clinicaltrials.gov shows that there have been 88 reported clinical trials using various formulation of GBE. Of the 88 trials, 66 have been concluded, and there are 30 Phase 3 or 4 trials. Most of these trials dealt with neural and cognitive disorders, where GBE has been shown to have clinical promise. For GBE beneficial effects in CVDs, 7 out of the 88 trials were concerned with vascular diseases, 4 with stroke, 4 with arteriosclerosis, 2 with coronary disease, 1 with hypertension, and 1 with atherosclerosis.

GBE has vasorelaxation effects in human subjects. GBE was able to dilate forearm blood vessels causing changes in regional blood flow without affecting BP levels in 16 healthy subjects (Mehlsen et al., 2002). A small trial performed in normal glucose-tolerant subjects to determine the effects of GBE on glucose-stimulated pancreatic beta-cell function found that the ingestion of GBE for three months can decrease SBP and DBP. In these individuals, fasting plasma insulin and CRP were increased (Kudolo, 2000). A double-blind, placebo-controlled, parallel design trial was performed in patients with peripheral artery disease aimed to assess the effects of the supplementation of 300 mg/day of EGb761 to treadmill walking time and cardiovascular measures. In older adult patients, EGb761 produced a modest non-significant increase in maximal treadmill walking time and flow-mediated vasodilation. The authors suggested that a longer duration might be needed to observe significant beneficial effects (Gardner et al., 2008).

Kuller el al. used the Ginkgo Evaluation of Memory Study (GEM) to assess CVD as a secondary outcome. The GEM study was a double-blind trial that randomized 3069 participants whose ages were over 75 years to 120 mg of EGb761 twice daily (240 mg/day) or placebo. Data indicated that EGb761 did not affect the originally assessed primary outcome—the development of dementia or Alzheimer's disease (DeKosky et al., 2008). Also, there were no differences in the incidence of myocardial infarction, angina pectoris, or stroke between the GBE and placebo groups. After 6 years of monitoring, the study concluded that GBE does not reduce total CVD mortality or CVD events (Kuller et al., 2010).

Several clinical trials to assess the protective effects of GBE in CVDs are still ongoing. A 12-weeks randomized, double-blind, phase 3 clinical trial aimed to further evaluate the safety and efficacy of Rinexin^®^ (Cilostazol 100mg, *Ginkgo biloba* leaf extract 80mg) which is widely used as an anti-platelet agent for the treatment of peripheral artery disease (NCT03318276; clinicaltrials.gov). Most recently, efficacy and safety of *Ginkgo biloba* pills for CHD patients with impaired glucose regulation will be assessed in a Phase 4 randomized, double-blind, placebo-controlled clinical trial (NCT03483779; clinicaltrials.gov). Twelve patients will be recruited for a test period of 58 weeks. Pills of five different GBEs will be to administered three times a day (Sun M. et al., 2018).

The therapeutic effect GBE appears to be more evident in combination with modern medicine. The analysis of 23 randomized clinical trials (involving 2,529 patients) showed that when combined with routine Western medicine, GBE was more effective at the relief of angina pectoris as compared to the routine medicine alone (Sun et al., 2015). In addition, due to its platelet aggregation inhibitory effects, the combination of GBE and modern medicine was reported to posses beneficial effects against acute cerebral ischemia. In that study, platelet aggregation was found to be significantly lower in patients treated with ticlopidine and EGb 761 as compared with patients treated with ticlopidine alone (Hong et al., 2013). Combination of EGb 761 also had increased therapeutic effect in patients with uncontrolled diabetes. Indeed, a randomized controlled trial showed that the combination of EGb 761 with metformin is more effective than metformin alone in improving the outcomes of patients with uncontrolled T2DM (Aziz et al., 2018).

The GBE therapeutic potential in managing CVDs has not been always clinically observed. Using data obtained from the GEM study database, Brinkley et al. concluded that GBE does not reduce BP or the incidence of hypertension in older men and women (Brinkley et al., 2010). In accordance, another study reporting the analysis of 9 randomized clinical trials (1012 hypertensive patients) concluded that more rigorous trials are needed to draw a conclusion on the efficacy of GBE in managing hypertension (Xiong et al., 2014).

Based on these and other studies, the efficacy of GBE, despite being reported in many studies, is best documented and observed when combined with other known medications for the management of CVDs.

### Safety, Toxicity, and Side Effects of *G. biloba*

Taken orally at the typical dosage, GBE may cause mild adverse effects, principal among which are mild gastrointestinal upset, headache, dizziness, constipation, and allergic skin reactions. Higher dosages, however, can result in restlessness, diarrhea, nausea, vomiting, and weakness (Diamond and Bailey, 2013). Noticeably, the therapeutic employment of this herb is also linked to adverse cardiovascular events. Fifteen published case reports described a temporal association between GBE intake and serious bleeding events, including intracranial bleeding (Bent et al., 2005), an effect that may be attributed to the platelet-activating factor antagonism exerted by ginkgolides, bilobalides, and other constituents present in the extract (Izzo and Ernst, 2009). These major bleeding events, including subarachnoid and intracranial hemorrhage, have been mostly described during the concomitant use of gingko and antiplatelet and/or anticoagulant medications (Matthews, 1998). Therefore, it is recommendable to stop GBE intake at least 2 weeks before surgical procedures. Always because of its anti-platelet properties, it has been suggested that GBEs (including seeds and leaves) should be used with caution during pregnancy, particularly around labor, and during lactation (Dugoua et al., 2006). Several case reports described cardiac adverse events associated with *Ginkgo biloba* leaf extracts. For example, 2 weeks GBE intake (40 mg, three times daily) has been reported to develop ventricular arrhythmia in a 49-year-old subject with good health (Cianfrocca et al., 2002), a symptom resolved upon the discontinuation of GBE supplementation (Cianfrocca et al., 2002).

A randomized placebo-controlled, double-blind pilot study of GBE reported more ischemic stroke and transient ischemic attack cases in the GBE group as compared with the placebo. The study lasted 42-month 118 cognitively intact subjects randomized to standardized GBE or placebo and its aim was to measure the effect of GBE on cognitive decline (Dodge et al., 2008). Another case report, attributed the frequent nocturnal palpitations reported by a 35-year old woman taking GBE supplementation to GBE (Russo et al., 2011). In addition to clinical trials, *Ginkgo biloba* safety has also been assessed *in vivo* in rats. Dietary intake of GBE (0.5% extract) for 4 weeks has been reported to significantly reduce heart rate and blood flow velocity in tail arteries of old spontaneously hypertensive (SH) rats as compared to the control group (Tada et al., 2008). Thus, in the elderly population with hypertension, the use of GBE may need to be assessed for effects on heart rate (Mei et al., 2017).

Furthermore, some of the components (ginkgolic acids) of EGb761 have been reported to elicit severe allergic reactions. However, this allergic reaction is not present as long as the carboxylic acid group of ginkgolic acids is intact (Chan et al., 2007). Yet, contact with *Ginkgo biloba* plants is associated with severe allergic reactions, including erythema and edema (Chiu et al., 2002)

Food poisoning by *Ginkgo biloba* seeds has been reported in Japan and China, where the main symptoms were convulsion, vomiting, and loss of consciousness. The poisoning is primarily due to the neurotoxic compound 4′-O-methylpyridoxine (MPN, also known as ginkgotoxin) which interferes with pyridoxine (vitamin B_6_) metabolism, leading to serious neurological manifestations including neurotoxicity, seizures, and loss of consciousness (Wada et al., 1988; Wada, 2005). Ginkgotoxin is found in the ginkgo leaf at very low amounts. However, GBE is unlikely to contain this toxic component as ginkgotoxin is standardized to be too low in the extract (Arenz et al., 1996).

Several reports have described that GBE induces cytochrome P450 (CYP) in humans, shedding light on potential interactions between GBE and conventional drugs. *Ginkgo biloba* is known to decrease the plasma concentrations of omeprazole, ritonavir, and tolbutamide. It can interact with antiepileptics, acetylsalicylic acid, diuretics, ibuprofen, risperidone, rofecoxib, trazodone, and warfarin (Izzo and Ernst, 2009).

Considering that GBE is widely used in a plethora of diseases combined with the paucity of data from animal studies regarding GBE toxicity and carcinogenicity, the National Institutes of Health (NIH) has performed a 2-year and 3-month toxicity and carcinogenicity study of GBE in B6C3F1/N mice and F344/N rats using different doses of GBE. The GBE used contained 24% flavonol glycosides and 6% terpene lactones, along with no more than five ppm ginkgolic acids. The study was performed by NIH National Toxicology Program (NTP) and concluded that GBE might elicit toxic and cancer-related consequences in rodents. The carcinogenic effects reported were stomach ulcers, organ modification including carcinogenic activity in the liver, liver and thyroid gland hypertrophy, liver hyperplasia, and hyperkeratosis (National Toxicology Program, 2013; Rider et al., 2014). These reports raised concerns about the safety of GBE. Following the NTP report, the International Agency for Research on Cancer (IARC) reported in 2014 that there is inadequate evidence in humans for the carcinogenicity of GBE (Grosse et al., 2013). Following this report, clinical and genomic safety of IDN 5933/Ginkgoselect^®^Plus, a standardized GBE, was assessed in elderly subjects using a randomized placebo-controlled clinical trial. The treatment group was given 120-mg IDN 5933 twice-daily for 6 months. No adverse clinical effects or increase of liver injury markers were reported in the treatment group. Genomic testing revealed that there is no difference in micronucleus frequency or DNA breaks between the treated and placebo groups. The expression of genes known to be modulated in early carcinogenesis (c-*myb, p53*, and *ctnnb1* [β-catenin]) was not significantly different between groups at the beginning or the end of the study (Bonassi et al., 2018). Taken together these results support the safety of IDN 5933 at the used doses for a duration of 6 months. Overall, there is still controversy about the safety of GBE for long-term use in human subjects and additional well-designed clinical trials that assess the safety and efficacy of GBE are much needed.

## Ganoderma lucidum

**Figure 4:**
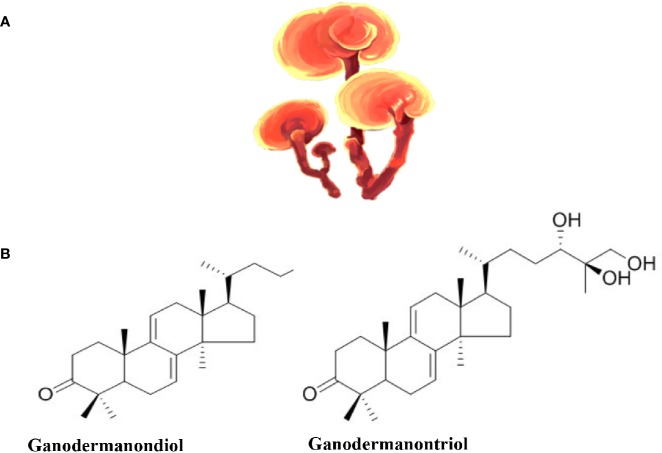
*Ganoderma lucidum*. **(A)** *Ganoderma lucidum* (from https://pngtree.com/freepng). **(B)** Examples of the chemical structure of two Triterpenes from *Ganoderma lucidum*.

*Ganoderma lucidum*, also known as “lingzhi” or “reishi,” is a mushroom (**Figure 4**) whose different parts (mycelia, spores, and fruit body) are used to make different forms of commercial *G. Lucidum* for their medicinal benefits. Commercially, *G. Lucidum* is available as powders, dietary supplements, tea, among other forms. Historically, *G. Lucidum* medicinal use has been wide spread in Asian countries (mainly in China, Japan, and Korea) for more than 2000 years. Later, it was introduced to Western societies (Ahmad, 2018). Hot water or ethanol can be used to extract the bioactive compounds from the fruiting bodies, the mycelia, or the spores of the mushroom (Heleno et al., 2012). A wide array of bioactive compounds exist in *G. Lucidum* that include triterpenes (**Figure 4**), polysaccharides, nucleosides, steroids, fatty acids, alkaloids, proteins, peptides, amino acids, and inorganic elements (Ahmad, 2018).

Immunomodulation, anti-oxidation, liver protection, anti-proliferation, and anti-angiogenesis are among the various properties that *Ganoderma lucidum* bioactive compounds possess individually or synergistically (Sanodiya et al., 2009). The triterpenoids have hepatoprotective, anti-hypertensive, hypo-cholesterolemic, anti-histaminic, anti-tumor, and anti-angiogenic effects. In addition, *Ganoderma lucidum* triterpenoids have anti-platelet aggregation and complement inhibition effects. It should be noted that *G. Lucidum* is the only source of triterpene fatty acids called ganoderic acids. Of the 200 bioactive compounds that have been identified in *Ganoderma lucidum* extracts, ganoderic acids A, B, and C have hypoglycemic effects (Hikino et al., 1985; Tomoda et al., 1986), while ganoderic acids F, B, D, H, K, S, and Y most likely have hypotensive effects (Morigiwa et al., 1986). *Ganoderma lucidum* polysaccharides (β-d-glucans) harbor anti-tumor properties due to their immunomodulation and anti-angiogenesis effects. They also exhibit anti-oxidant protective effects against free radicals and can decrease mutagen induced cell damage (Boh et al., 2007). *Ganoderma lucidum* β-d-Glucan polysaccharides were also identified as the active component in the polysaccharide peptide (PsP) usually found in *Ganoderma lucidum* extracts. There is no agreed dosage for *G. lucidum*, however, commonly used doses vary between 1.5 and 9 g of dried extract per day (Klupp et al., 2016).

### *G. lucidum* at the Bench: Mechanism of Action in CVDs

As stated earlier, antioxidants are very important when it comes to the prevention of atherosclerosis. *G. lucidum* can act as an antioxidant; in the model organism *Caenorhabditis elegans, G. lucidum* protected *C. elegans* against paraquat and heavy metal-induced oxidative stress *via* the diet restriction pathway and the mTOR/S6K signaling pathway, respectively (Cuong et al., 2019). *G. lucidum* has been found to protect human lymphocyte DNA from hydrogen peroxide-induced oxidative damage (Shi et al., 2002). It was also found that *G. lucidum* regulates the expression of Nrf2 which in turn regulates antioxidants genes such as HO-1, GST, NQO-1; *G. lucidum* ethanol extract enhanced the phosphorylation of Nrf2 which upregulated HO-1 in C2C12 myoblasts (Lee Y. et al., 2016). Thus, the antioxidant power in *G. lucidum*, which is important for the prevention of atherosclerosis, can act on different signaling pathways.

Elevated BP can be detrimental to heart function. Three peptides (QLVP, QDVL, and QLDL), that can inhibit ACE activity and called ACE inhibitory peptides (ACEIPs), were extracted from *G. lucidum* and can be used to treat hypertension. QLVP can inhibit ACE by its interaction with Gln242 and Lys472 of ACE. QLVP also enhanced Angiotensin 1-mediated phosphorylation of eNOS and reduced mRNA and protein expression of the vasoconstrictor peptide endothelin-1 in HUVECs (Wu et al., 2019).

Selenium-enriched *G. lucidum* polysaccharide (Se-GLP) extracts have protective effects against oxidative damage in a mouse model of heart reperfusion injury. Se-GLP significantly reduced the ischemic reperfusion injury-induced serum levels of MDA as well as the levels of the proinflammatory molecule intercellular adhesion molecule-1. Heart and serum levels of the antioxidant enzymes SOD, CAT, and GSH-Px and the levels of GSH as well as total antioxidant capacity were rescued by Se-GLP (Shi et al., 2010). In another study, the protective effects of Ganoderic acid A, from *G*. *lucidum* extracts, was found to activate the PI3K/AKT signaling pathway causing increased proliferation and decreased apoptosis of rat H9c2 cardiomyocytes exposed to hypoxic injury, a phenomenon mediated by the activation of miR-182-5p and reduction of PTEN expression (Zhang et al., 2018). Similarly, a preclinical study using transverse aortic constriction mice as a model of pressure overload-caused cardiomyopathy reported improved cardiac function following treatment with spore oil extracted from *G. Lucidum* (Xie et al., 2016).

More research needs to be conducted on the possible mechanisms and signaling pathways that *G. Lucidum* bioactive derivatives employ to elicit their beneficial effects in order to get a more comprehensive sense of how these substances work.

### *In Vivo* Preclinical Evaluation of *G. lucidum*

To study the cardioprotective effects of *G. lucidum* extract, global ischemia and reperfusion of isolated and perfused rat hearts were used. As a preventative treatment, 400 mg/kg body weight of *G. lucidum* extract was delivered to rats for 15 days. It was found that this treatment halted the necrotic death of the rat cardiomyocytes and reduced the reperfusion contracture (Lasukova et al., 2015). Interestingly, *G. lucidum* extract supplementation attenuated diastolic dysfunction and prevented irreversible cardiomyocyte damage (Lasukova et al., 2008).

Diabetes mellitus is a metabolic disease that has been correlated with a high incidence of CVDs, partly because high blood glucose levels can cause vascular damage (Klein et al., 2002; Fox et al., 2008; Shah et al., 2008). Thus, a supplement that can help manage diabetes would be of great benefit to decrease CVD risk. Thirty-five Wistar rats were supplemented with 50, 150, and 300 mg/kg of PsP from *G. lucidum* extracts. PsP induced endothelial repair process with 300 mg/kg being the most efficient dose. The findings showed that vascular damage was improved by PsP treatment in a rat model of T2DM (Heriansyah et al., 2019).

A diet high in fats is known to be a contributing risk factor to the development of CVDs (Fleming, 2002) and weight loss is an essential measure for the prevention of CVDs in obese people (Sowers, 2003). The water extract of *G. lucidum* can reduce the body weight, inflammation, and insulin resistance in HFD-fed mice (Chang et al., 2015). In another study, *G. lucidum* spores (GLSP) at a dose of 1 g/day supplemented orally for 4 weeks were used in adult male Sprague-Dawley rats. GLSP decreased total cholesterol and triglycerides in diabetic rats as well as attenuated the levels of oxidative stress; it is worth noting that there was an upregulation of genes related to lipid metabolism—acyl-CoA oxidase 1 (ACOX1), acetyl-CoA carboxylase (ACC), and Insig-1/2 gene expression. ACOX1 was activated more than 5-folds in the GLSP-treated diabetic rats which indicates an accelerated beta-oxidation of lipids in these rats (Wang et al., 2015).

Dyslipidemia is one of the major risk factors for CVDs (Miller, 2009). In experimental animal studies, *G. lucidum* showed anti-hyperlipidemic effects by lowering plasma total cholesterol, LDL, and triglyceride levels (Chen et al., 2005). Hydrogen peroxide free radicals are usually elevated during dyslipidemia, and thus the risk of atherosclerosis, so antioxidants are needed to prevent blood vessels damage. A study conducted in high cholesterol diet-supplemented Wistar rats that received 50, 150, and 300 mg/kg bodyweight of PsP derived from *G. Lucidum* found that PsP acted as a potent antioxidant. In addition, PsP may prevent the atherogenesis process in the context of dyslipidemia with the optimum dose being 300 mg/kg bodyweight (Wihastuti and Heriansyah, 2017). Another study found that polysaccharides extracted from *G. Lucidum* significantly reduced the body-weight increases of mice fed an HFD suggesting its role as a hypolipidemic substance. In addition, it exhibited antioxidant and antiapoptotic effects in the HFD-fed mice (Liang et al., 2018).

With regard to regulation of high BP, a study conducted on adult male hypertensive rats that received *G. lucidum* water extracts intra-gastrically for seven weeks found that the experimental rats BP was reduced to a level comparable to that of rats on losartan (angiotensin II receptor antagonist) (Shevelev et al., 2018)

### *G. lucidum* to the Clinic

Antioxidants are potentially therapeutic substances that can be used to prevent atherosclerosis as well as a variety of other diseases. Several *in vitro* and *in vivo* preclinical studies have shown that *G. lucidum* constituents possess antioxidant activities, but evidence for such activities in human subjects was lacking. Watchtel Galor et al. performed a follow-up study on the effects of *G. lucidum* supplementation on an array of parameters that included antioxidant biomarkers status and CHD risk. A double-blinded, crossover, placebo-controlled intervention study of 18 healthy people was conducted. There was an enhancement of plasma total antioxidant markers status as well as an improvement of CHD biomarkers after 10 days of supplementation. More importantly, there was no renal, liver, or DNA toxicity (Wachtel-Galor et al., 2004). To test *G. Lucidum* antioxidant potential, a crossover human intervention study was conducted on seven healthy people, and it was found that the plasma total antioxidant power was enhanced after the administration of a single dose of *G. Lucidum* extract. Lymphocytes harvested following blood collection, however, showed no signs of enhanced DNA repair (Wachtel-Galor et al., 2010). *G. lucidum* PsP was also examined for its antioxidant properties. A clinical trial was conducted with 37 high risk and 34 stable angina patients that were given PsP 750 mg/day for 90 days. PsP proved to be a potent antioxidant in the context of atherosclerosis in both the high angina risk and stable groups. PsP supplemented groups showed increased SOD, decreased MDA levels, and reduced counts of circulating endothelial cells (CEC) and endothelial progenitor cells (EPC) (Sargowo et al., 2018).

A double-blind, randomized, multicentered study was done to evaluate the effects and safety of *G. lucidum* polysaccharides on patients with CHD. Eighty-eight patients that constituted the experimental group were given *G. lucidum* polysaccharides for 12 weeks. The polysaccharides significantly enhanced the wellbeing of the patients evidenced as an improvement of their major symptoms (chest pain, shortness of breath, palpitations), and a decrease in BP and serum cholesterol levels (Gao et al., 2004). Another study found that PsP from *G. lucidum* had anti-inflammatory effects and vascular EC protection in patients with ST-elevation myocardial infarction and non-ST-elevation myocardial infarction with risk factors of dyslipidemia (Sargowo et al., 2019).

A randomized clinical trial was conducted on the use of *G. lucidum* for the treatment of cardiovascular risk factors of the metabolic syndrome. It was reported that using *G. lucidum* (3 g/d) for 16 weeks had no effect on glycosylated hemoglobin (HbA1c) and fasting plasma glucose (FPG). Further, consumption of *G. lucidum* was associated with the increased risk of a subset of mild events including headache, fatigue, and gastrointestinal events (Klupp et al., 2016).

None of the human studies reported above had any serious side effects thus highlighting *G. lucidum* as a potential therapeutic and preventative substance in the context of certain CVDs.

In a study examining 37 patients with high risk for atherosclerosis and on an ongoing conventional CVD medication, *G. lucidum* PsP was able to reduce the levels hs-CRP, IL-6, and TNF-α, as well as the levels of MDA (Widya et al., 2016). Another study involving 34 stable angina pectoris patients showed that administration of PsP in combination with previous medication is able to significantly reduce CECs and EPCs (markers for endothelial vascular injury) as well as the levels of total cholesterol (Ubaidillah et al., 2016).

The efficacy of *G. lucidum* in CVDs was tested in a prospective, double-blind, placebo-controlled trial. Eighty-four volunteers with T2DM and metabolic syndrome were supplemented with extracts of *G. lucidum, G. lucidum* with *Cordyceps sinensis*, or placebo in order to manage cardiovascular risk factors. Results from the study evidenced that *G. lucidum* failed to provide benefit against CVDs in patients with the metabolic syndrome (Klupp et al., 2016). Likewise, the analysis of five trials (398 total volunteers), concluded that *G lucidum* was not effective in treating elevated BP (Klupp et al., 2015).

Taken together, these data suggest that *G. lucidum* is most effective in providing cardiovascular protection when combined with conventional CVDs medications.

### Safety, Toxicity, and Side Effects of *G. lucidum*

Numerous conducted studies and investigations pointed toward the safety of *G. Lucidum*. In both male and female rodents, *G. lucidum* shows no signs of toxicity even at a dose of up to 5000 mg/kg of body weight. No animal mortality was reported either (Smina et al., 2011). When compared to doxorubicin (a typical DNA intercalating agent used in chemotherapy), *G. lucidum* extracts were shown to act indirectly on DNA, thus adding more assurance to its safety (Gurovic et al., 2018).

Hemostatic parameters, platelet and global hemostatic functions, were not affected when a dose of 1.5 g/d of *G. lucidum* extract was given to healthy human volunteers for 4 weeks. In addition, the use of *G. lucidum* did not cause bleeding problems in the healthy subjects (Kwok et al., 2005). However, in another study, Tao and Feng volunteered 15 healthy subjects and 33 atherosclerotic patients and found that any dose above 3000 mg/day can inhibit platelet aggregation. As a result, caution is advised when supplementing *G. lucidum* to patients with low platelet count or patients that will undergo surgical procedures (Tao and Feng, 1990).

Safety of polysaccharides extracted from the fruiting body of *G. lucidum* was evaluated in Wistar rats and the results indicated no evidence of abnormal clinical symptoms, death or significant differences in body weight and food intake. No significant differences were found in the hematology value, clinical chemistry value and organ/body weight ratio. Additionally, no mutagenicity was detected in Kunming mice (Zhang et al., 2016).

However, high doses of *G. lucidum* polysaccharides modulated immune responses. The polysaccharides enhanced the primary immune response to sheep red blood cells but did not have significant effects on the phagocytic function or macrophages (Zhang et al., 2016). The *G. lucidum* polysaccharides-modulated immune responses were tested in another study where 16 participants supplemented with 2 g of *G. lucidum* extract twice daily took part in a 10-day controlled trial. In this study, there were no differences in CD4, CD8, and CD19 levels in the blood, though CD56 did increase, without achieving statistical significance, but it returned to baseline levels 10 days after intake of the extract (Wicks et al., 2007).

A 12-week trial performed on 23 dyslipidemic and mild hypertensive volunteers revealed that Lingzhi (1.44 g/day) had no effect on several clinical chemistry parameters as compared with the placebo. However, symptoms such as headache, influenza/running nose were found, although considered not clinically significant (Chu et al., 2012).

Whether these changes that are induced by *G. lucidum* have any clinical value remains to be addressed in future clinical trials especially after long-term administration of escalating doses of *G. lucidum* extracts to assess long-term safety. Further studies are still needed to examine the toxicity, side effects, and safety of *G. lucidum* for human consumption.

## Gynostemma pentaphyllum

**Figure 5:**
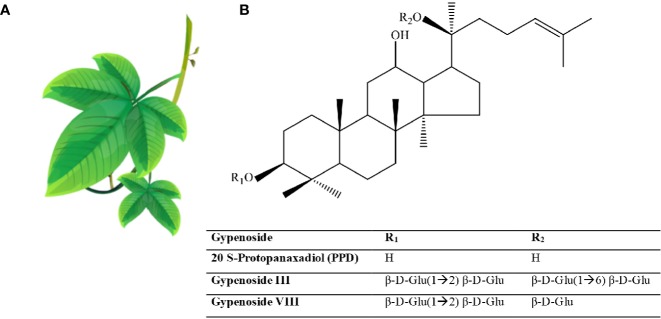
*Gynostemma pentaphyllum*. **(A)** *Gynostemma pentaphyllum* (Source https://pngtree.com/freepng). Examples of the chemical structure of *Gynostemma pentaphyllum* Gypenoside that are usually synthesized 20 S-Protopanaxadiol (PPD). **(B)** example of some chemical structures of Gypenosides.

*Gynostemma pentaphyllum* (**Figure 5**), also known as Jiaogulan, is a herbaceous climbing vine. Originating in south China and now widely distributed in South and East Asia (Li et al., 2016), it is found in subtropical China, Japan, Myanmar, and India (Chen and Gilbert, 2006), growing near rivers and in the shade of forests that surround Yangtze River basin and the southern areas of China (Chen, 1995). It can be found as a health supplement in beverages, biscuits, face washes, and bath oils (Li et al., 2016). The herb has a low level of genetic diversity and a high level of variation among populations (Zhang et al., 2019).

Phytochemical analysis found that *G. pentaphyllum* extracts contain gypenoside saponins, flavonoids, polysaccharides, and amino acids (Zheng, 2004; Nookabkaew et al., 2006; Yan et al., 2013). Its biological effects can range from antimicrobial (Srichana et al., 2011), antioxidant (Muller et al., 2012), anticancer (Schild et al., 2010), anti-inflammatory (Wong et al., 2017), antidiabetic (Yeo et al., 2008; Huyen et al., 2012), antilipidemic (la Cour et al., 1995), and neuroprotective (Keilhoff et al., 2017) to anti-obesity effects (Gauhar et al., 2012; Park S. et al., 2014). It has been used to treat hepatitis and hypertension (Lin et al., 2000). Among the various bioactive compounds, dammarane-type triterpene saponins (gypenosides or gynosaponins) are major (Kim and Han, 2011).

### *G. pentaphyllum* at the Bench: Mechanism of Action in CVDs

As already mentioned, antioxidants are important when it comes to the prevention of atherosclerosis. Four flavonoids—namely quercetin-3-O-(2″,6″-di-α-L-rhamnosyl)-β-D-galactopyranoside, quercetin-3-O-(2″,6″-di-α-L-rhamnosyl)-β-D-glucopyranoside, quercetin-3-O-(2″-α-L-rhamnosyl)-β-D-galactopyranoside, and quercetin-3-O-(2″-α-L-rhamnosyl)-β-D-glucopyranoside—with potent antioxidant effects against DPPH and OH free radicals, *in vitro*, were found in the extracts of *G. pentaphyllum*. These flavonoids also exhibited cytoprotection against AAPH-induced oxidative damage in pig kidney LLC-PK1 cells by suppressing the increase of MDA, and limiting the decrease of SOD and GSH (Lin et al., 2019). In another study, flavonoids from *G. pentaphyllum* were extracted and tested on human lung carcinoma A549 cells. It was found that the flavonoids protected A549 cells against hydrogen peroxide-induced oxidative damage by increasing the expression levels members of the endogenous antioxidant system including SOD, GSH, Nrf2, NQO1, and HO-1 (Wang et al., 2018). Another study evaluated the antioxidant potential of one *G. pentaphyllum* component, the phytoestrogen gypenoside XVII, it was found that the phytoestrogen alleviated atherosclerosis *via* the ERα-mediated PI3K/Akt pathway (Yang et al., 2017). A prior study evaluated the effects of gypenosides of *G. pentaphyllum* on hydrogen peroxide-induced oxidative damage in bovine pulmonary artery ECs. The gypenosides protected the ECs from oxidative injury further suggesting its potent antioxidant activity as well as its prospective use as a preventative supplement against atherosclerosis (Li and Lau, 1993).

Inflammation can contribute to the onset of atherosclerosis and other CVD risk factors, hence, reducing inflammation can act as a protective factor in CVDs. The gypenoside XLIX (Gyp-XLIX) from *G. pentaphyllum* has been studied for its anti-inflammatory properties. Gyp-XLIX inhibited LPS- and TNF-α-induced NF-κB activation in THP-1 monocytes and in HUVECs. Gyp-XLIX inhibition of NF-κB activation appears to be through a PPAR-α-dependent pathway (Huang et al., 2006). On the other hand, contradictory results were reported by Aktan et al., where *G. pentaphyllum* gypenosides attenuated NF-κB activation. In fact, the gypenosides extracted from *G. pentaphyllum* could suppress NO production by inhibiting iNOS activity and levels in murine macrophages. Gypenoside-mediated decrement of iNOS protein expression turned out to be mediated by the inhibition of NF-κB activation (Aktan et al., 2003). In a different study, Tanner et al. showed that *G. pentaphyllum* could elicit beneficial effects on vascular function by acting as an inducer of eNOS (Tanner et al., 1999).

Attenuating lipid accumulation may decrease CVDs incidence. A study assessed the role of ombuine, a dual agonist of PPAR-α and PPAR-δ/β in lipid metabolism. Ombuine or mbuin-3-O-β-d-glucopyranoside, a flavonoid from *G. pentaphyllum*, were applied to HepG2 cells. Ombuine-stimulated HepG2 cells had activated PPAR-α and PPAR-δ/β, transcription factors that enhance lipolysis. Ombuine-mediated activation of PPAR-α and PPAR-δ/β significantly reduced intracellular concentrations of triglyceride and cholesterol as well as decreased lipogenic gene expression witnessed as decreased levels of sterol regulatory element binding protein-1c and stearoyl-CoA desaturase-1. These findings further our understanding of how *G. pentaphyllum* may be involved in lipid metabolism (Malek et al., 2013).

In a study that evaluated the role of total flavonoids of *G. pentaphyllum* on apoptosis in cardiomyocytes of neonatal rats, it was found that hypoxia-reoxygenation (H/R)-cardiomyocytes had an increased protein expression of apoptosis-associated Fas/FasL genes. Flavonoids of *G. pentaphyllum* could protect cardiomyocytes against H/R injury by decreasing the production of TNF-α and downregulating the protein levels of Fas/FasL genes leading to inhibition of myocyte apoptosis (Le et al., 2007).

### *In Vivo* Preclinical Evaluations of *G. pentaphyllum*

Gypenosides are *G. pentaphyllum* key components with the ability to help prevent atherosclerosis. The anti-atherosclerotic effects of a mixture (HG) of *Fermentum rubrum* Hongqu and *G. pentaphyllum* gypenosides were investigated in Wistar rats. The study results revealed that the HG mixture had anti-atherosclerotic effects that were better than statin, simvastatin, treatment highlighting the anti-atherosclerotic potential of *G. pentaphyllum* (Gou et al., 2018). In addition, HG alleviated oxidative stress biomarkers by restoring antioxidant defense components and decreasing the serum levels of anti-inflammatory cytokines in male Sprague-Dawley rats with fatty liver disease. The HG mixture in this study displayed athero-protective characteristics (Gou et al., 2016).

Emphasizing the reported *G. pentaphyllum* gypenoside anti-inflammatory and antioxidant capabilities, Yu et al. demonstrated their beneficial effects in an ischemia–reperfusion injury rat model where they were found to inhibit apoptosis. Ischemia–reperfusion injury has detrimental outcomes in CHD. Yu et al. found that in ischemia reperfusion injured-rats, the administration of gypenosides decreased apoptotic rates as well as improved cardiac function. Gypenosides inhibited ER-stress and apoptosis through the blockade of the CHOP pathway and the activation of PI3K/Akt pathway (Yu et al., 2016b). In a different study, it was demonstrated that pretreatment with gypenosides limited the infarct size and relieved ischemia reperfusion-induced pathological changes in the myocardium, also in an ischemia reperfusion injury rat model. Additionally, left ventricle function was preserved. Molecularly, gypenosides pre-treatment reduced oxidative stress and restored the antioxidant machinery in the myocardium. The cardio-protective effects were also evidenced by the preservation of mitochondrial function in myocytes. In this regard, the maintenance of the mitochondrial membrane integrity inhibited the release of cytochrome c from the mitochondria into the cytosol. This further demonstrated the role of gypenosides from *G. pentaphyllum* as a cytoprotective agent against acute myocardial infarction and reperfusion injury (Yu et al., 2016a).

As elucidated to earlier, diabetes is positively correlated with the development of CVDs. The effect of *G. pentaphyllum* extracts on fasting blood sugar levels in diabetic mice was assessed. It was found that the extracts had inhibitory effects on α-glucosidase activity while affecting the protein expression of GLUT2 which highlights its potential to manage diabetes (Wang et al., 2019).

Attenuating obesity, a risk factor for developing CVDs, may decrease CVD incidence*. G. pentaphyllum* is largely used for the management of diseases such as hyperlipidemia, fatty liver, and obesity in China. *G. pentaphyllum* was found to affect lipid metabolism and elicit anti-hyperlipidemic effects by elevating the levels of phosphatidylcholine and decreasing the levels of trimethylamine N-oxide in the plasma and liver of rats (Wang et al., 2013). In addition, Gauhar et al. studied the effects of heat processed ethanol extracts of *G. pentaphyllum* on obese mice. They found that this extract decreased obesity in *ob/ob* mice by activating the AMP-activated protein kinase (AMPK) pathway. This study suggested a possible mechanism for fat-loss as well as a potential for the use of *G. pentaphyllum* as a weight-loss supplement (Gauhar et al., 2012). Gypenosides anti-hyperlipidemic effects were examined in rats with poloxamer P407-induced hyperlipidemia. Gypenosides at 250 mg/kg of body weight was orally administered to hyperlipidemic rats. Four and 12 days of gypenosides administration reduced plasma triglycerides levels by 53% and 85%, respectively. Similarly, total cholesterol levels were decreased by 10% and 44%, respectively. Interestingly enough, results were similar to atorvastatin cholesterol-lowering statin drugs. Additionally, LDL levels were reduced and HDL levels were increased by gypenoside, which also reversed the poloxamer P407 inhibition of lipoprotein lipase activity. This shows a promising therapeutic potential of *G. pentaphyllum* for lowering high triglyceride and cholesterol levels during acute hyperlipidemia (Megalli et al., 2005). In accordance, extracts from the plant also decreased triglyceride levels and LDL levels in obese Zucher rats (Megalli et al., 2006). By employing a new extraction technique, Lee et al. described some biological activity of a *G. pentaphyllum* extract with a higher content of gypenoside L (1.8% w/w), gypenoside LI (1.4% w/w), and ginsenoside Rg3 (0.15% w/w) (Lee et al., 2019b). While HFD-fed mice showed significant clinical effects such as increases in body weight, fat mass, white adipose tissue, and adipocyte hypertrophy as compared to the control group, the GPE-treated group failed to show them. GPE treatment also reduced serum levels of triglyceride, total cholesterol, and LDL-cholesterol, without affecting HDL-cholesterol. Mechanistically, the clinically observed GPE effects appeared due to increased AMPK activation and suppressed adipogenesis by decreasing the mRNA levels of CCAAT/enhancer binding protein-α (C/EBPα), PPARγ, SREBP-1c, PPARγ coactivator-1α, fatty acid synthase, adipocyte protein 2, and sirtuin 1, and increased levels of carnitine palmitoyltransferase and hormone-sensitive lipase (Lee et al., 2019b).

### *G. pentaphyllum* to the Clinic

In general, there are few human trials that addressed *G. pentaphyllum* extract therapeutic effects or safety. A search on clinicaltrials.gov shows that there are only four studies that use *G. pentaphyllum* extracts. One of the studies is on obese patients and three are on diabetes mellitus patients.

Actiponin, an extract of *G. pentaphyllum*, is a dietary supplement used for weight loss in obese individuals. During an interventional study, 80 randomized Korean participants took part in a double blind, parallel study for 12 weeks where the experimental group took Actiponin at a dose of 450 mg/day. The experimental group lost weight with no adverse effects, as compared to the placebo group (Park S. et al., 2014).

In another study, 1 mg/kg of the water extract of *G. pentaphyllum* was given to 44 patients with CVDs and 56 healthy individuals and the platelet aggregation was studied. It was revealed that the water extract elicited significant inhibition of the aggregation of platelets. This means that there is potential for the use of this supplement to prevent cardio-cerebrovascular diseases while being cautious not to give these supplements to patients who suffer from low platelet count or bleeding disorders (Juan and Shanzhang, 1995).

Some studies suggest there is a link between anxiety disorders and an increased risk of developing a CVD (Fan et al., 2008; Vogelzangs et al., 2010; Seldenrijk et al., 2011; Batelaan et al., 2014). For this reason, it is of interest to reduce anxiety in order to decrease the risk of developing CVD in predisposed individuals. It was shown that *G. pentaphyllum* ethanol extract had anti-anxiety effects on mice exposed to chronic stress (Choi H. et al., 2013). This finding was replicated in a double-blind, placebo-controlled clinical trial that had 72 healthy Korean individuals under chronic stressful conditions. Thirty-six participants were given 200 mg of *G. pentaphyllum* ethanol extract, twice a day for 8 weeks. The supplementation reduced the experimental group anxiety without any adverse drug effects suggesting its potential as a safe anti-anxiety supplement (Choi et al., 2019).

Although the proven pharmacological effects of *G. pentaphyllum* in *in vitro* studies and *in vivo* animal studies may not necessarily translate well into efficacy human subjects, there are positive studies. For instance, a set of studies conducted in T2DM patients supplemented with *G. pentaphyllum* tea showed improvements in insulin sensitivity and glycemia with no adverse side effects (Huyen et al., 2010; Huyen et al., 2013). In one of the trials, 24 drug-naïve T2DM patients were randomized to take either 6 g daily of *Gynostemma pentaphyllum* tea or placebo tea, during 1- week period. The authors measured FPG, insulin levels, and HbA1c levels before, during, and after the treatment. The study showed a prompt improvement of glycemia and insulin sensitivity, and suggested that *Gynostemma pentaphyllum* tea could be an effective, and safe approach to treat T2DM patients (Huyen et al., 2010). The same authors followed up with another study that used the same study design but enrolled only 16 drug-naïve T2DM patients. The authors measured the same parameters and came to the same conclusion that *Gynostemma pentaphyllum* tea exerted antidiabetic effects by improving insulin sensitivity (Huyen et al., 2013). These results were confirmed by another study where *G. pentaphyllum* was used together with sulfonylurea (Huyen et al., 2012). Thus far, the current data indicate that *G. pentaphyllum* is quite efficient at improving insulin sensitivity and blood sugar levels if administered solely and that its efficacy may be enhanced when combined with other medications.

### Safety, Toxicity, and Side Effects of *G. pentaphyllum*

In a study evaluating the toxicity of *G. pentaphyllum* extract on female Sprague-Dawley rats, a single dose of up to 5000 mg/kg of the extract was given and subchronic toxicity tests were performed with 1000 mg/kg/day for 90 days. No rat death occurred nor did any signs of toxicity arise. Blood chemistry values, though statistically different from the control group, were within normal ranges in rats. Thus, no mortality nor abnormalities have risen from the *G. pentaphyllum* extract treatment (Chiranthanut et al., 2013). In addition, no toxicity or mortality was reported upon long-term administration of a dose up to 750 mg/kg body weight of *G. pentaphyllum* in rats (Attawish et al., 2004).

A Phase I clinical trial was conducted to evaluate the safety of *G. pentaphyllum* whereby three groups of healthy volunteers were administered 50, 200, and 400 mg twice daily with water extract of *G. pentaphyllum* for two months. No major immune adverse events such as significant changes in natural killer cell activities, number of CD3+, CD4+, and CD8+ cells, were reported. No biochemical parameters were significantly affected either. Such doses of *G. pentaphyllum* were deemed to be safe (Chavalittumrong et al., 2007). In another clinical trial, 537 bronchitic patients were treated three times a day with *G. pentaphyllum* (2.5–3 g, prepared as tablets or capsules). Adverse side effects that included vomiting, abdomen tension, diarrhea (or constipation), dizziness, blurred vision, and tinnitus effects were seen in a small number of patients. Notably, these symptoms were mild and did not stop the patients from taking the *G. pentaphyllum* extract (Razmovski-Naumovski et al., 2005). A very recent randomized, double-blind, placebo-controlled clinical trial utilizing *G. pentaphyllum* extract in 72 healthy adults revealed no adverse side effects of the ingestion of the ethanolic extract of *G. pentaphyllum* (Choi et al., 2019). Overall, consumption of *G. pentaphyllum* seems to be safe at the doses required to observe a therapeutic effect.

## Conclusion

**Figure 6:**
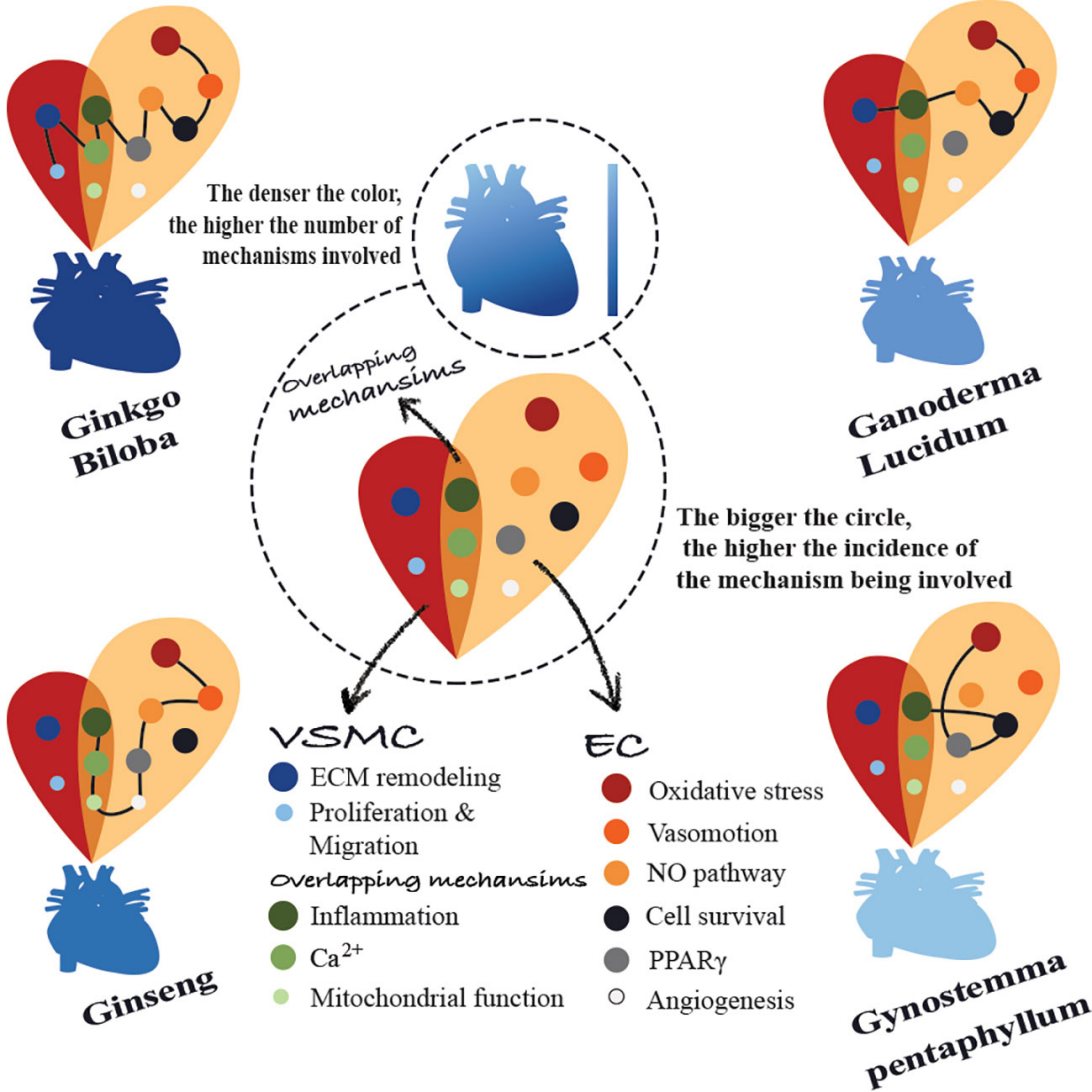
Herbal therapies in the context of CVDs. Herbal preparations can exert protective effects by ameliorating the pathological effects exerted by CVDs risk factors. The herbal extracts can attenuate endothelial dysfunction and/or VSMC alterations by acting as, vasodilators, ROS scavengers, anti-oxidants, anti-inflammatory, anti-apoptotic, anti-hypertrophic, or anti-proliferative agents. This achieved through mechanisms that act in ECs only, VSMCs only, or through overlapping mechanism that act in both ECs and VSMCs. In ECs, herbal preparations can increase NO availability, decrease mitochondrial dysfunction and/or metabolic abnormalities as well as enhance angiogenesis. This can decrease the incidence of atherosclerosis and hypertension, which in return can decrease the risk of CVDs development. In VSMCs, the herbal extracts can modulate ECM deposition as well as cell migration, proliferation, and cell shape changes. VSMC, vascular smooth muscle cell; ECM, extracellular matrix; EC, endothelial cell; NO, nitric oxide; PPARY, peroxisome proliferator-activated receptor-gamma.

Despite the abundance of knowledge regarding CVDs, CVD prevalence continues to be on the rise. Thus, there is an immediate demand for new safe, effective, and relatively cheap drug candidates. Mounting evidence obtained from *in vitro* and *in vivo* studies suggests that the four traditionally used medicinal plants discussed in this review significantly modulate key cellular, molecular, and metabolic mechanisms that control both CVDs pathogenesis and pathophysiology (**Figure 6**). Here are presented the recent findings, advances, and studies describing the therapeutic values of these plants in the context of several CVDs. Current evidence demonstrates that these herbal medicines have potent therapeutic properties and can ameliorate pathological conditions associated with CVDs (**Table 2** and **Figure 6**). However, clear clinical therapeutic benefits have not yet been secured. As such, these herbal treatments cannot be safely recommended as an alternative therapeutic medicine. In fact, the safety and toxicity of some of these plants have recently raised potential concerns (*eg. Gingko Biloba*). We conclude that better-designed studies and future clinical trials involving larger sample sizes are needed to investigate the role of different medicinal plants and their underlying mechanisms in the context of CVDs. Above all, the future clinical trials should address the safety and toxicity of these herbal remedies.

**Table 2:** Summary of the mechanisms of action of the four discussed plants.

Plant		Mechanism of action	References
***Ginseng***	**Bioactive/active fractions:** Rb1, Rg1, Rg3, Re, and Rd **Commonly used extracts:** *Panax ginseng, Panax notoginseng*, *Panax quinquefolium L., Panax japonicas*	**Improves lipid profile** By acting as an agonist of PPAR **Controls hypertension and improve endothelial function** By Inhibition of ACE By reducing adrenal catecholamines levels, elevating NO and cGMP levels By activating Ca2+-gated potassium channels **Controls inflammation** By inhibiting AP‐1 and NF‐κB By reducing COX‐2, IL‐6, IL‐1β, TNF‐α, CD68, MCP-1 and MMP levels **Ameliorates oxidative stress** By exhibiting free radical scavenging and metal ion chelating abilities By promoting enhanced expression of antioxidant proteins, such as Nrf2 and HO-1 **Ameliorates mitochondrial dysfunction** **Modulates angiogenesis** By decreasing VEGF-A and FGF-2 levels	(Kim et al., 1999b; Keum et al., 2003; Park et al., 2005; Persson et al., 2006; Shin Y. et al., 2013; Park J. et al., 2014; Lee H. et al., 2016; Deng et al., 2017; Singh et al., 2017; Lee et al., 2019a)
***Gingko Biloba***	**Bioactive/active fractions:** Ginkgolides classified into either A, B, C, J, or M types **Commonly used extracts:** EGb761	**Improves lipid profile** By decreasing PPARs levels **Controls hypertension and improves endothelial function** By decreasing ACE activity, activating cholinergic pathways, limiting LPS-induced proliferation of VSMCs By decreasing ICAM‐1 and VCAM‐1 expression, decreasing phosphorylation of Akt/FoxO3a By restoring eNOS activity, decreasing iNOS expression and consequently elevating NO levels **Controls inflammation** By suppressing TLR-4 expression By decreasing MMP-1, MCP-1, TNF-α, IL-6, or IL-1β **Ameliorates oxidative stress** By decreasing NOX activity and level, activating endogenous Akt/Nrf2 antioxidant stress pathway By increasing levels of HO-1, SOD and GSH-Px By reducing the phosphorylation of MAPKs **Prevents hypertrophy** By activating M2 muscarinic receptors/NO pathway By decreasing calcium overload and inhibiting the Na^+^/Ca^2+^ exchanger **Prevents apoptosis** By decreasing caspase 3 and pro-apoptotic Bax expression and increasing anti-apoptotic Bcl-2 expression	(Akiba et al., 2007; Lin et al., 2007; Mansour et al., 2011; Liu et al., 2013; Liou et al., 2015; Mesquita et al., 2017; Abdel-Zaher et al., 2018; Huang et al., 2018; Chen et al., 2019).
***Ganoderma lucidum***	**Bioactive/active fractions:** ganoderic acids A, B, C, D, F, H, K, S, and Y, β-d-Glucan polysaccharides **Commonly used extracts:** Polysaccharide peptide (PsP) Ganoderma lucidum	**Controls hypertension and improves endothelial function** By inhibition of ACE, enhancing Angiotensin 1-mediated phosphorylation of eNOS By reducing the levels of vasoconstrictor peptide Endothelin-1 **Improves lipid profile** By upregulating lipid metabolism (ACOX1 and ACC) **Ameliorates oxidative stress and inflammation** By enhancing phosphorylation of Nrf2 which upregulates HO-1, GST, NQO-1, SOD, CAT, GSH-Px and GSH By decreasing the levels of MDA and ICAM, and regulation of mTOR/S6K signaling pathways **Reduces necrosis** By decreasing the levels of creatine phosphokinase	(Hikino et al., 1985; Tomoda et al., 1986; Shi et al., 2002; Shi et al., 2010; Lasukova et al., 2015; Wang et al., 2015; Wihastuti and Heriansyah, 2017; Cuong et al., 2019; Wu et al., 2019)
***Gynostemma pentaphyllum***	**Bioactive/active fractions:** dammarane-type triterpene saponins (gypenosides or gynosaponins) **Commonly used extracts:** Actiponin, Ombuine	**Improves lipid profile** By activation of PPAR-α and PPAR-δ/β, decreasing the levels of sterol regulatory element binding protein-1c and stearoyl-CoA desaturase-1 By activation of the AMPK pathway **Ameliorate oxidative stress and decreases apoptosis** By decreasing the levels of MDA, increasing the levels of SOD, GSH, Nrf2, NQO-1 and HO-1 By downregulating Fas/FasL, blocking CHOP pathway By regulating the activation of PI3K/Akt pathway **Controls inflammation** By decreasing LPS- and TNF-α-induced NF-κB through regulating PPAR-α	(Huang et al., 2006; Megalli et al., 2006; Malek et al., 2013; Yu et al., 2016b; Yang et al., 2017; Wang et al., 2018; Lin et al., 2019)

PPAR, peroxisome proliferator-activated receptor; ACE, acetylcholinesterase; NO, nitric oxide; AP‐1, activator protein 1; NF‐κB, nuclear factor kappa-light-chain-enhancer of activated B cells; COX, cyclooxygenase; IL, interleukin; TNF, tumor necrosis factor; CD, cluster of differentiation; MCP, monocyte chemoattractant; MMP, matrix metalloproteases; Nrf2, nuclear factor erythroid-2-related factor 2; HO-1, hemeoxygenase-1; VEGF, vascular endothelial growth factor; FGF, fibroblast growth factor; VSMCs, vascular smooth muscle cells; ICAM, intercellular adhesion molecule; VCAM, vascular cell adhesion molecule; Akt, protein kinase B; FoxO3a, forkhead box O; eNOS, endothelial nitric oxide synthase; iNOS, inducible nitric oxide synthase; TLR, toll-like receptor; NOC, nicotinamide adenine dinucleotide phosphate oxidase; SOD, superoxide dismutase; GSH-Px, glutathione peroxidase; MAPK, mitogen-activated protein kinase; ACOX1, peroxisomal acyl-coenzyme A oxidase 1; ACC, acetyl-CoA carboxylase; GST, glutathione S-transferase; NQO-1, NAD(P)H dehydrogenase (quinone); GSH, glutathione; MDA, malondialdehyde; mTOR, mammalian target of rapamycin complex; S6K, S6 kinase; AMPK, AMP-activated protein kinase; CHOP, C/EBP homologous protein.

## Author Contributions

AS ideated and wrote the manuscript. DT, HP, TN, and GN contributed to manuscript writing and editing. SAH, HH, and SH contributed to manuscript writing and figures drawing. AE and GP contributed to manuscript ideation, revision, and editing

## Funding

This work has been made possible thanks to grants (Ager S.O.S.) and (fondo di Ateneo per la ricerca 2019) to GP and Qatar University grant (IRCC-2019-007) to GN and GP.

## Conflict of Interest

The authors declare that the research was conducted in the absence of any commercial or financial relationships that could be construed as a potential conflict of interest.
